# Inhibition of Angiopoietin-2 Production by Myofibrocytes Inhibits Neointimal Hyperplasia After Endoluminal Injury in Mice

**DOI:** 10.3389/fimmu.2018.01517

**Published:** 2018-07-02

**Authors:** Daxin Chen, Ke Li, El-Li Tham, Lin-Lin Wei, Ning Ma, Philippa C. Dodd, Yi Luo, Daniel Kirchhofer, John H. McVey, Anthony Dorling

**Affiliations:** ^1^Division of Transplantation Immunology and Mucosal Biology, Faculty of Life Sciences and Medicine, King’s College London, Guy’s Hospital, London, United Kingdom; ^2^Medical Research Centre, Second Affiliated Hospital, Jiao Tong University School of Medicine, Xi’an, China; ^3^Department of Cardiology, Guangzhou First People’s Hospital, Guangzhou Medical University, Guangzhou, China; ^4^Department of Early Discovery Biochemistry, Genentech Inc., South San Francisco, CA, United States; ^5^Faculty of Health and Medical Sciences, School of Bioscience and Medicine, University of Surrey, Guildford, United Kingdom

**Keywords:** fibrocyte, angiopoietin-2, tissue factor, thrombin, CXCL12, intimal hyperplasia, vascular diseases

## Abstract

Fibrocytes are myeloid lineage cells implicated in wound healing, repair, and fibrosis. We previously showed that fibrocytes are mobilized into the circulation after vascular injury, including the immune-mediated injury that occurs after allogeneic transplantation. A common response to inflammatory vascular injury is intimal hyperplasia (IH), which, alongside vascular remodeling, results in progressive loss of blood flow, downstream ischemia, and end-organ fibrosis. This forms the pathological basis of transplant arteriosclerosis and other diseases including post-angioplasty re-stenosis. In investigating whether fibrocytes contribute to IH, we previously showed that subpopulations expressing smooth muscle actin and CD31 are recruited to the site of injury and accumulate in the neointima. Expression of tissue factor (TF) by these “CD31+ myofibrocytes” is needed for progressive neointimal expansion, such that TF inhibition limits the neointima to a single layer of cells by day 28 post-injury. The aim of this study was to determine pathophysiological mediators downstream of TF that contribute to myofibrocyte-orchestrated IH. We first show that myofibrocytes make up a significant component of the neointima 28 days following injury. Using a previously defined adoptive transfer model, we then show that CD31+ myofibrocytes get recruited early to the site of injury; this model allows manipulations of the adoptively transferred cells to study how IH develops. Having confirmed that inhibition of TF on adoptively transferred cells prevents IH, we then show that TF, primarily through the generation of thrombin, induces secretion of angiopoietin-2 by myofibrocytes and this directly stimulates proliferation, inhibits apoptosis, and induces CXCL-12 production by neointimal cells, including non-fibrocytes, all of which promote progressive IH *in vivo*. Prior incubation to inhibit angiopoietin-2 secretion by or block TIE-2 signaling on adoptively transferred fibrocytes inhibits IH. These novel data indicate that angiopoietin-2 production by early recruited myofibrocytes critically influences the development of IH after vascular injury and suggest new therapeutic avenues for exploration.

## Introduction

Chronic vascular disease commonly involves intimal hyperplasia (IH) and remodeling of arteries, leading to progressive reduction in blood flow, tissue fibrosis, and eventual organ failure. IH is characterized by progressive accumulation of cells expressing α-smooth muscle actin (SMA) in the intima: the precise origin of these cells has been extensively debated, but several studies in mice (1) and humans (2) suggest that some of these cells can be bone marrow (BM)-derived (3).

Working in both wire-induced endoluminal injury and allogeneic aortic transplantation models (4), we previously showed that a BM-derived cell type had a critical influence on the development of IH (5, 6). Subsequently, we identified that a monocyte-derived cell type was an early constituent of the neointima. We characterized these as CD45+ cells expressing CD31, TIE-2, VEGF-R2, and E-selectin (5), a phenotype consistent with previous descriptions of a neointimal cell type (7). Importantly, these CD45+ cells also expressed CD34 and collagen-1, identifying them as a fibrocyte subset. We also showed that fibrocytes were mobilized into the peripheral blood of mice post-injury and post-transplantation, accounting for approximately 45% of all CD34+ cells mobilized.

This offered an opportunity to develop adoptive transfer models, whereby circulating CD34+ cells isolated by magnetic bead-depletion from post-injury donor mice, and containing fibrocytes, are transferred to secondary host mice either on the day of injury or 7 days post-transplantation, depending on the model (4, 5, 8). Under these conditions, using cells harvested from donor mice expressing yellow fluorescent protein in all cells, we showed that the transferred CD34+ cells are recruited early to the injured host vessel wall. CD34+ cells from wild-type (WT) donor mice (but not CD34-negative cells) can transfer IH into a strain of host mice that are resistant to IH (4, 6), confirming that circulating CD34+ cells mobilized post-injury in the donor have an important role in orchestrating the intimal proliferative response following injury or allotransplantation. Moreover, donor CD34+ cells transferred into hosts either 2 weeks post-injury or 3 weeks post-transplantation get recruited to the neointima, suggesting that continuous recruitment of fibrocytes from the circulation may have an important influence on the progressive accumulation of cells in the neointima (5).

Neointimal fibrocytes express tissue factor (TF), and two strains of transgenic (Tg) mice expressing a membrane-tethered inhibitor of TF-FVIIa-FXa [human tissue factor pathway inhibitor (hTFPI)] on different cells (6, 9, 10) have been useful tools to dissect the importance of coagulation proteases in these models. Moreover, the hTFPI can be used to isolate and track cells *in vivo*.

In one of the strains, called “CD31-TFPI-Tg” mice, hTFPI expression is found in all CD31+ cells (10), but within the CD34+ fraction, only SMA+ fibrocytes (myofibrocytes) express the hTFPI on the cell surface (8). In this strain, although these CD31+ myofibrocytes are recruited to the vessel wall post-injury, no IH develops, and adoptive transfer of CD34+ cells from these mice to injured WT mice inhibits the development of IH, a phenotype that is due to the expression of the hTFPI transgene, as an inhibitory anti-hTFPI antibody reverses the protection offered by the hTFPI (8).

In this paper, we investigated the molecules downstream of TF and thrombin-mediated signaling, focusing on the role and importance of angiopoietin-2. Our data provide novel insights into the cellular and molecular basis of IH and should provide a foundation for exploring novel pathophysiological and therapeutic avenues in translational research.

## Materials and Methods

### Animals and Experimental Models

Wild-type mice (C57BL/6 from Harlan Olac Ltd., Bicester, UK), heterozygous CD31-TFPI-Tg (10), and ROSA-enhanced yellow fluorescent protein (EYFP) (5) mice, were bred and maintained at King’s College London. All genetically modified animals have been maintained for more than 10 generations on a WT background. All procedures were approved by the UK Home Office.

#### Wire-Induced Endoluminal Carotid Artery Injury

Mice weighing 25–30 g (*n* = 6 per group) were anesthetized by intraperitoneal injection of 0.1 ml/10 g solution composed of 1 ml Hypnorm solution (0.315 mg fentanyl/ml and 10 mg fluanisone/ml) (VetaPharma Ltd., Leeds, UK), 1 ml Hypnovel solution (5 mg Midazolame/ml) (Roche, Garden City, UK), and 2 ml dHO_2_. Surgery was performed using a dissecting microscope (Stemi SV 6, ZEISS, Germany). A 0.015-inch angioplasty guide wire (Cook Incorporated, IN 47404, USA) was introduced into the exposed left common carotid *via* the external carotid artery and withdrawn/reinserted three times to denudate endothelial layer. The external carotid artery was ligated after removing the wire. After confirming restoration of normal blood flow, animals were allowed to recover. To block the function of human TFPI in CD31-Tg mice, some animals from each strain (*n* = 6 each group) were administrated with 80 ng/g anti-human TFPI in 50 µl saline (Enzyme Research Laboratories, Swansea, UK) or isotype control mAb at a same dose by tail vein immediately after wire-induced injury.

#### Adoptive Transfer of CD34+ Cells After Vascular Injury

CD34+ cells were purified from donor mice 2–4 days after injury. Briefly, murine peripheral blood was collected into 1 mM EDTA or 3.2% sodium citrate and diluted with 2% FCS-PBS. Pelleted cells were re-suspended in ACK buffer and left at room temperature for 25 min, before washing to remove platelets. Purified CD34+ cells were isolated using magnetic beads (Miltentyi Biotech, Surrey, UK) as previously described (5, 6) and had an average purity of 95%. 1 × 10^6^ CD34+ cells, or purified fractions were injected into a second mouse *via* a tail vein on the day of injury. In some experiments, CD34+ cells were incubated with 100 µg/ml rat anti-mouse TF antibody (11) or 10 µg/ml anti-mouse TIE-2 antibody (Abcam, Cambridge, UK) and equal dose of isotype control antibodies, or 250 pmols siRNA/ml (see below) for 1 h in a 24-well plate, immediately prior to injection.

### Morphometric Analysis and Immunohistology

Carotid arteries were embedded in optimum cutting temperature compound (OCT) (VWR International, Dorset, UK) before cross-sectioning. Morphometric analysis was performed after staining with the Accustain Elastin Stain kit (Sigma) and evaluation on an Olympus U-ULH microscope (Olympus Optical Co Ltd., Tokyo, Japan). Medial and neointimal area were determined with Image-Pro Plus TM software version 4.0 (Media Cybernetics, Silver Spring, MD, USA). At least three random sections were examined from each of six wire-injured arteries, by an investigator blinded to the identity of the sections. For immunofluorescence (IF) analysis, carotid arteries were excised and embedded in OCT (VWR), and cut into 5 µm sections before fixing in methanol for 60 min at −20°C. Frozen sections were immersed in 1% BSA(Sigma)-PBS for 30 min and then incubated overnight at 4°C with the following antibodies: goat anti-angiopoietin-2 (Santa Cruz biotechnology Inc.), mouse monoclonal anti-collagen 1 (as a marker of fibocytes), rabbit anti-CXCR4, rabbit anti-CXCL12 (all from Abcam), mouse anti-human TFPI (Enzyme Research Laboratories, Swansea, UK), and rat anti-mouse CD31(BD). All stained sections were mounted in Vectashield mounting medium with DAPI (Vector Laboratories). Sections were examined by a Leica DM-IRBE confocal microscope (Leica, Wetzlar, Germany) equipped with Leica digital camera AG and a confocal laser scanning system with excitation lines at 405, 488, 543, and 560 nm at magnifications 10×/0.40CS and 20×/0.70IMM (Leica, Planapo, Wetzlar, Germany). Images were processed using Leica-TCS-NT software associated with the Leica confocal microscope. For analysis of the neointimal area occupied by specific stains, data were collected from three random sections from each of six arteries, with the investigator blinded to the identity. For all molecules, control sections were stained with isotype-matched antibodies to confirm the specificity of staining (see Figure S1 in Supplementary Material).

### Thrombin Generation, Chemokine, and Angiopoietin Production

CD34+ cells (2 × 10^4^ per well in a 96-well plate) were washed and suspended in Dulbecco modified Eagle medium (DMEM; Sigma-Aldrich MO, USA) containing 10 nmol/l Factor X (FX) with or without 10 nmol/l factor VIIa (FVIIa) at 4°C. After 15 min, pre-prepared 10 nmol/l factor Va (FVa) and 0.5 µmol/l prothrombin (all from Enzyme Research Laboratories) in HEPES-buffered saline (Life Technologies, Grand Island, NY, USA) were added. At defined times, aliquots of the reaction mixture were transferred into Tris-EDTA buffer with the chromogenic substrate S-2238 (Chromagenix, Milan, Italy) to assess thrombin generation. Absorbance at 405 nm was converted to concentrations using purified standards control assays. To assess production of angiopoietin-2 and CXCL12 under these conditions, the assay was terminated after 20 min by washing the cells five times with PBS before re-culturing in DMEM containing 2% FCS for 24 h to obtain supernatant. To assess angiopoietin-2 and CXCL-12 production after thrombin stimulation, 2 × 10^4^ CD34+ cells were seeded on a round glass coverslip in a 24-well plate, serum starved for 24 h, following which varying concentrations of thrombin (0–100 nmol/l) (Enzyme Research Laboratories, Swansea, UK) were added, with or without an anti-TIE-2 antibody (Abcam) before incubation in Iscove modified Dulbecco medium (IMDM; Sigma-Aldrich, MO, USA) supplemented with 2% fetal bovine serum (FBS; StemCell Technology, Grenoble, France) for 5 days.

To assess PAR-induced cell signaling, starved cells were treated with 0–20 µM of either PAR-1, PAR-2, or PAR-4 antagonists (Peptides International, Louisville, KY, USA) for 30 min before stimulation with FVIIa + FX or FVIIa + FX + FII at indicated doses; or cells were stimulated with 0–100 µM of PAR-1, PAR-2, or PAR-4 agonist (Peptides International); or cells were treated first with or without 50 µM mitogen-activated protein kinase inhibitor PD98059, 10 µM p38-MAPK inhibitor SB203580, 20 µM NF-kB inhibitor SN50, and 1 µM of the S6K1 inhibitor (All from Merck Millipore, Hertfordshire, UK) for 30 min and then stimulated with 10 µM of PAR-1, PAR-2, or PAR-4 agonist. 24 h after cell treatments, angiopoietin-2 concentration was determined in supernatants. In some assays, cells were first treated with the siRNA for angiopoietin 2 or the fluorescein conjugated control siRNA, using 2 × 10^5^ CD34+ cells per well were seeded in a six-well tissue culture plate (see below).

Angiopoietins-1 and -2 and CXCL12 were quantified using commercially available ELISA kits (Cusabio Biotech, DE19711, USA) according to the manufacturer’s protocol. The minimum detectable dose is less than 0.1 pg/ml for mouse angiopoietin-1 and 0.08 pg/ml for mouse angiopoietin-2. The same procedure was used to analyze plasma levels.

### siRNA Transfection

siRNA for angiopoietin 2 (sc-39294), a pool of 3 target-specific 19-25 nt siRNAs designed to knockdown gene expression, and the fluorescein conjugated control siRNAs (sc-36869) were purchased from Santa Cruz biotechnology Inc. Texas 75220, USA. 2 × 10^5^ CD34+ cells per well were seeded in a six-well tissue culture plate and transiently transfected using siRNA transfection reagents system (Santa Cruz Biotechnology Inc.) according to the manufacturer’s instructions. For *in vitro* use, 2 × 10^5^ cells per well were incubated with 50 pmols siRNA diluted into siRNA transfection medium. 24 h after transfection, cells were washed twice in serum-free medium and incubated with 50 nM thrombin for further incubation with the culture medium containing 2% FCS. 48 h later, cells were collected for assaying cell proliferation, fixed for analyzing cell phenotype and apoptosis and supernatants harvested for measuring angiopoietin 2 and CXCL12 productions. For *in vivo* use, see above.

### Cell Proliferation and Apoptosis

To determine cell proliferation, CD34 cells were seeded at 10^4^cells/well in 96-well plate. After the manipulations defined above, wells were pulsed with 0.037 MBq/ml (1 Ci/ml) [3H]-thymidine (Amersham Biosciences, Bucks, UK) for the last 18 h of culture and then harvested using a Micro 96 harvester (Skatron Instruments, Lier, Norway) and the incorporation of [^3^H] thymidine was determined using a liquid scintillation counter (1205 Betaplate; Wallac, Turku, Finland). Apoptosis was performed with an *in situ* cell death detection kit according to the manufacturer’s instructions (Roche Applied Science, Philadelphia, PA, USA). All analyses were performed in triplicate.

### Immunohistochemistry and Immunocytochemistry

CD34+ cells were spread on a glass coverslip (VWR International, Leuven, Belgium) and fixed with cold methanol for 10 min at −20°C, before incubation with specific antibodies at room temperature for 60 min. Cells were then incubated at room temperature for 60 min with following antibodies: mouse anti-human α-SMA conjugated with Cy3 (Sigma-Aldrich, St. Louis, MO, USA), rat anti-mouse CD31 (BD) and rabbit anti-collagen-1, rabbit anti-angiopoietin-1, rabbit anti-angiopoietin-2, rabbit anti-TIE-2, and rabbit anti-CXCL12 (all from Abcam). Second layer staining was with a goat anti-rat IgG-FITC, a goat anti-rabbit IgG-FITC, and a rabbit anti-goat IgG-FITC (all from Sigma-Aldrich). Stained cells were mounted in Vectashield mounting medium with DAPI (Vector Laboratories), and captured by a Leica DMIRBE confocal microscope (Leica, Wetzlar, Germany) as above.

### Statistical Analysis

Statistical analysis was performed with GraphPad Prism software. The Mann–Whitney test was used for comparison of two groups and the Kruskal–Wallis test for ≥3 groups. All data were reported as mean ± SEM. A value of *P* < 0.05 was considered statistically significant.

## Results

### Vailidity of Adoptive Transfer Model to Study the Role of Angiopoietin-2 Expressed by Myofibrocytes in Pathophysiology of IH

#### In WT Mice, Angiopoietin-2+ Myofibrocytes Recruited to Site of Endoluminal Injury Make Up a Significant Proportion of Neointima

**Figure 1:**
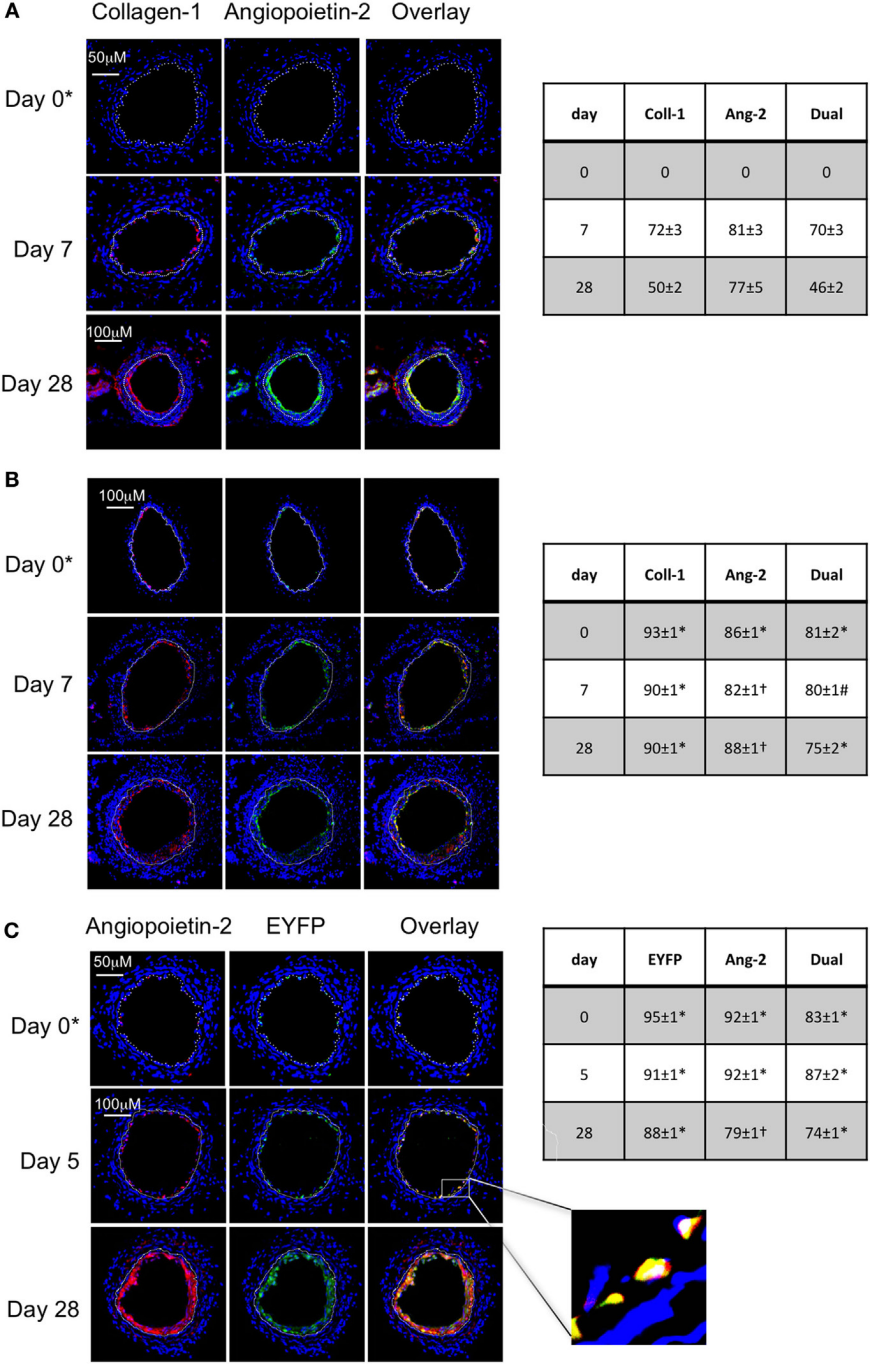
Comparison of phenotype of intimal hyperplasia in wild-type (WT) animals vs. WT after adoptive transfer of WT CD34+ cells immediately post-injury. Panels show immunohistology of representative sections through injured mouse carotid arteries harvested on days post-injury as indicated. All sections stained with DAPI (4,6 diamidino-2-phenylindole) nuclear stain (blue) and (red) anti-collagen-1 **(A,B)**, or angiopoietin-2 **(C)**, and (green) anti-angiopoietin-2 **(A,B)**. Yellow indicates co-localization. In **(C)**, the adoptively transferred cells came from enhanced yellow fluorescent protein (EYFP) mice, which spontaneously fluoresce as shown. The annotated white line defines the junction between neointima and media. **(A)** Injured mice received no adoptively transferred cells. *On day 0, sections harvested within an hour of injury. **(B)** Mice injected with cells from WT mice. **(C)** Mice injected with cells from EYFP mice. Expanded area illustrates that most of the early recruited cells are adoptively transferred, angiopoietin-2+ cells. Tables beside each panel describe summary of staining from all mice (*n* = 6), showing the proportion of the neointimal area that is positive for collagen-1, angiopoietin-2 or both or EYFP, angiopoietin-2 or both on days 0, 5/7, and day 28 (% ± SEM). Data derived from three random sections from each of the arteries from each mouse (see Materials and Methods). Subtracting the proportional area occupied by dual positive cells from the total area occupied by angiopoietin-2+ cells suggests the proportional area occupied by collagen-1-negative cells expressing angiopoietin-2. *cf WT (no adoptive transfer) at equivalent time: *p* < 0.001. ^†^cf WT (no adoptive transfer) at equivalent time, *p* = NS. ^#^cf WT (no adoptive transfer) at equivalent time *p* = 0.04. Experiments repeated at least twice.

Following wire-induced injury, we have previously shown that myofibrocytes are quickly mobilized into the circulation, reaching peak numbers by 2–3 days (5, 8). We confirmed in this new work that these cells get recruited to the luminal aspect of the injured vessel, so that collagen-1+ cells are easily detectable by day 7 and make up ≥50% of the neointimal area for up to 28 days post-injury (Figure 1A). Many of these cells co-expressed angiopoietin-2 (Figure 1A) but a significant proportion of the angiopoietin-2 expression was in collagen-1-negative, areas, suggesting that angiopoeitin-2 was expressed by cells in the neointima other than myofibrocytes. This data re-affirm that the response to endoluminal injury involves recruitment of myofibrocytes, that these comprise a significant proportion of the neointimal area 28 days post-injury, and that they express angiopoietin-2.

#### Adoptive Transfer of CD34 Cells Results in Recruitment of Donor Myofibrocytes to the Site of Injury

As previously described, CD34+ cells from WT mice were adoptively transferred into secondary host WT mice immediately following endoluminal injury. Collagen-1+ cells were detectable at the site of injury within several hours after injection (Figure 1B), occupied a slightly larger proportion of neointimal area as in WT mice without adoptive transfer, and co-expressed angiopoietin-2 (Figure 1B). Expression of angiopoietin-2 by non-fibrocytes was similar to that seen without adoptive transfer. Confirmation that the adoptively transferred cells were recruited to the neointima came from the transfer of CD34+ cells from EYFP mice (Figure 1C). These data indicate that the adoptive transfer of WT CD34+ cells results in IH that is broadly similar in phenotype to the basal disease developing after endoluminal injury in WT mice.

#### Confirmation That TF on Myofibrocytes Is Involved in IH and Associates With Angiopoietin-2 Expression

**Figure 2:**
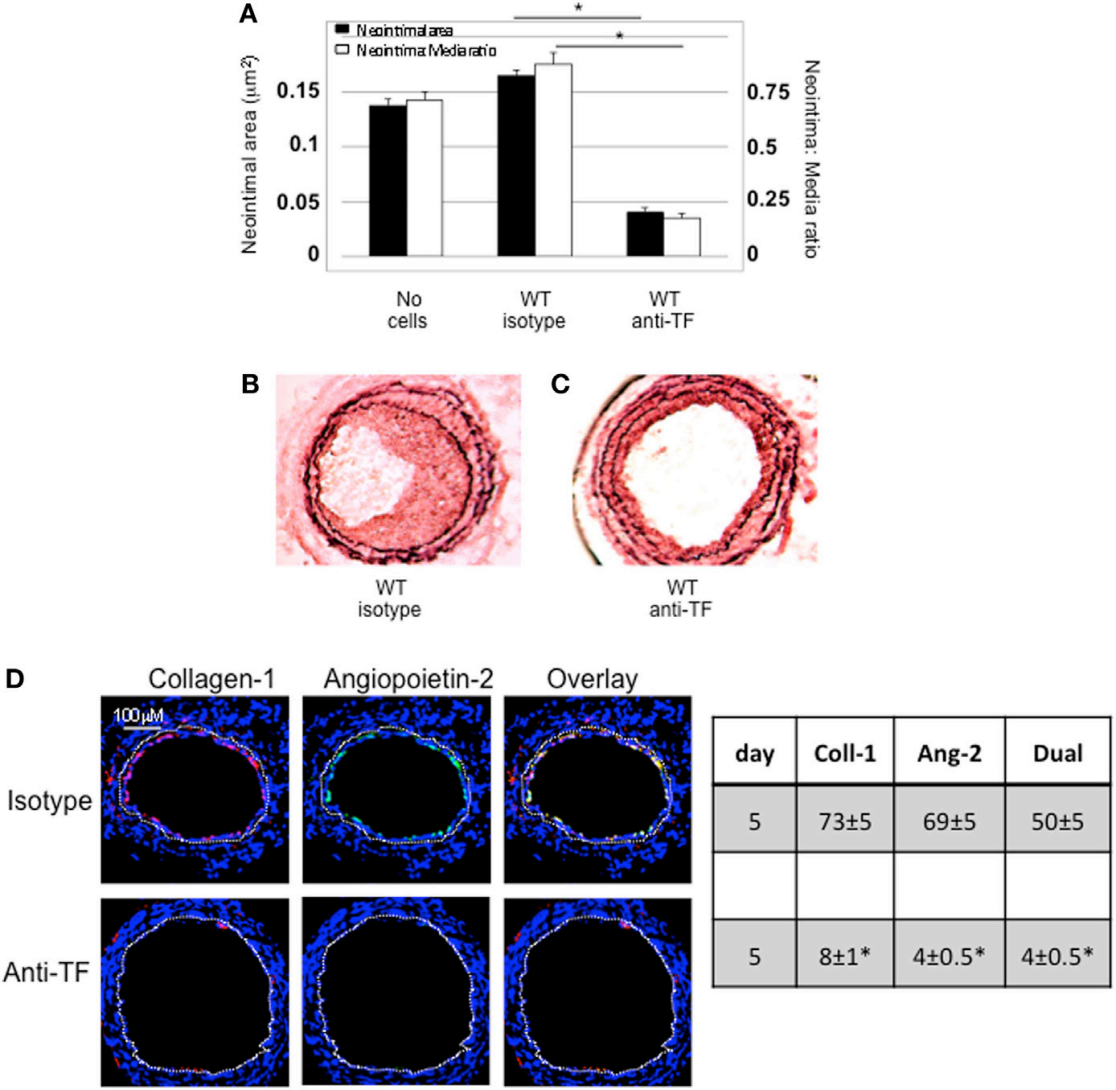
Pre-incubation of wild-type (WT) CD34+ cells with an inhibitory anti-TF antibody prior to adoptive transfer. **(A)** Neointimal area (left axis, black bars) and intima:media ratio (right axis, white bars) of vessels taken from WT animals 28 days post-injury compared to those in mice after adoptive transfer of 1 × 10^6^ WT CD34+ cells that were either pre-incubated with isotype control or an anti-TF antibody immediately prior to injury. Data derived from examination of three random sections from five different vessels. *p < 0.01. **(B,C)** Cross sectional images of WT carotid artery 28 days post-injury stained with elastin van Gieson’s stain after adoptive transfer of cells pre-incubated with isotype control antibody **(B)** or anti-TF antibody **(C)** prior to injury. **(D)** Panels show immunohistology of consecutive sections through injured mouse carotid arteries harvested on day 5 post-injury. All sections stained with DAPI (4,6 diamidino-2-phenylindole) nuclear stain (blue) and anti-collagen-1 (red) and (green) angiopoietin-2. Yellow indicates co-localization. The annotated white line defines the junction between neointima and media. Table besides panel **(D)** describes summary of staining from all mice (*n* = 6), showing the proportion of the neointimal area that is positive for collagen-1, angiopoietin-2, or both on day 28 (% ± SEM). Data derived from three random sections from each of the arteries from each mouse (see Materials and Methods). Subtracting the proportional area occupied by dual positive cells from the total area occupied by angiopoietin-2+ cells gives the proportion of neointimal collagen-1-negative cells expressing angopoietin-2. *cf WT with isotype control *p* < 0.001 Experiments repeated at least twice.

Wild-type CD34+ cells were incubated with an inhibitory anti-TF antibody prior to transfer into injured WT mice. IH was completely inhibited in these mice, compared to mice injected with cells incubated with isotype control antibody (Figures 2A–C). Pre-treatment with anti-TF antibody induced a significant reduction in the proportion of angiopoietin-2+ cells in the neointima, but also caused a significant reduction in the proportion of collagen-1+ cells recruited (Figure 2D).

Adoptively transferred CD34+ cells contain numerous subpopulations, and these data imply that inhibition of TF on all subpopulations has a significant impact on the recruitment of fibrocytes, akin to findings we have previously described in a second strain of transgenic mice (8), but not directly relevant to the question being addressed by this experimental work.

**Figure 3:**
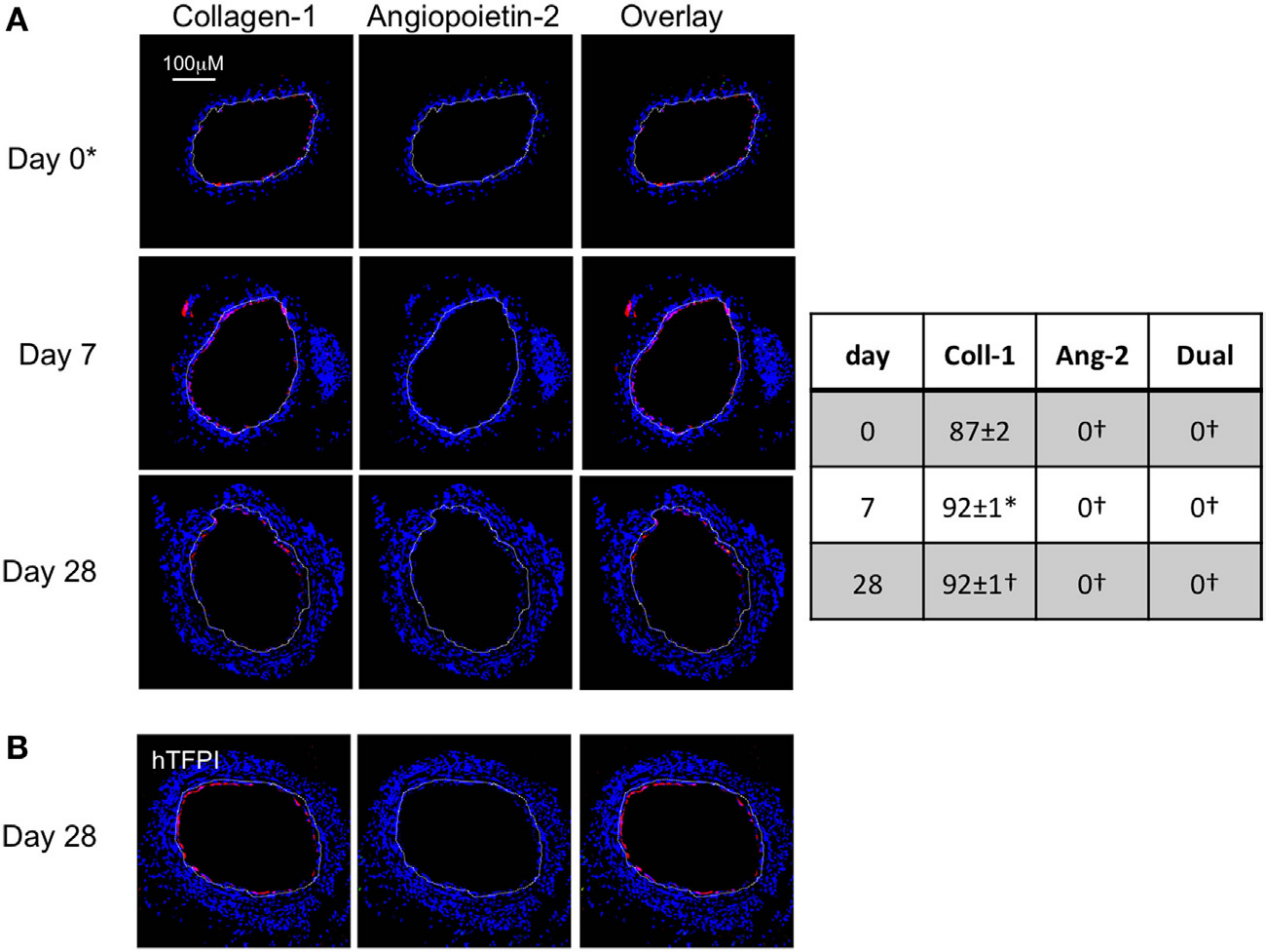
Adoptive transfer of CD31-TFPI-Tg CD34+ cells to wild-type (WT) mice immediately post-injury. Panels show immunohistology of representative sections through injured mouse carotid arteries harvested on days post-injury as indicated. All sections stained with DAPI (4,6 diamidino-2-phenylindole) nuclear stain (blue) and (red) anti-collagen-1 **(A)** or human tissue factor pathway inhibitor **(B)** and (green) anti-angiopoietin-2. Yellow indicates co-localization. The annotated white line defines the junction between neointima and media. *On day 0, sections harvested within an hour of injury. Table besides panel **(A)** describes summary of staining from all mice (*n* = 6), showing the proportion of the neointimal area that is positive for collagen-1, angiopoietin-2, or both on days 0, 7, and 28 (% ± SEM). Data derived from three random sections from each of the arteries from each mouse (see Materials and Methods). *cf WT cells (see Figure 1B) at equivalent time point *p* = 0.03. ^†^cf WT cells (Figure 1B) at equivalent time point *p* < 0.001. Experiments repeated at least twice.

To assess the impact of TF inhibition on myofibrocytes only, we adoptively transferred CD34+ cells from CD31-TFPI-Tg mice. These mice mobilize similar subpopulations of CD34+ cells into the circulation as WT mice, but constitutively express cell-surface hTFPI fusion protein only on myofibrocytes (8). We have previously shown that transfer of CD34+ cells from these mice inhibits the development of IH (8). After adoptive transfer, a significant proportion of the cells recruited to the site of injury are collagen-1+ but angiopoietin-2 negative (Figure 3A). The small amount of neointima present at day 28 contained a majority of collagen-1+, hTFPI+, and angiopoietin-2-negative cells (Figure 3B), confirming that failure to develop IH associated with absence of angiopoietin-2 expression in the recruited myofibrocytes.

**Figure 4:**
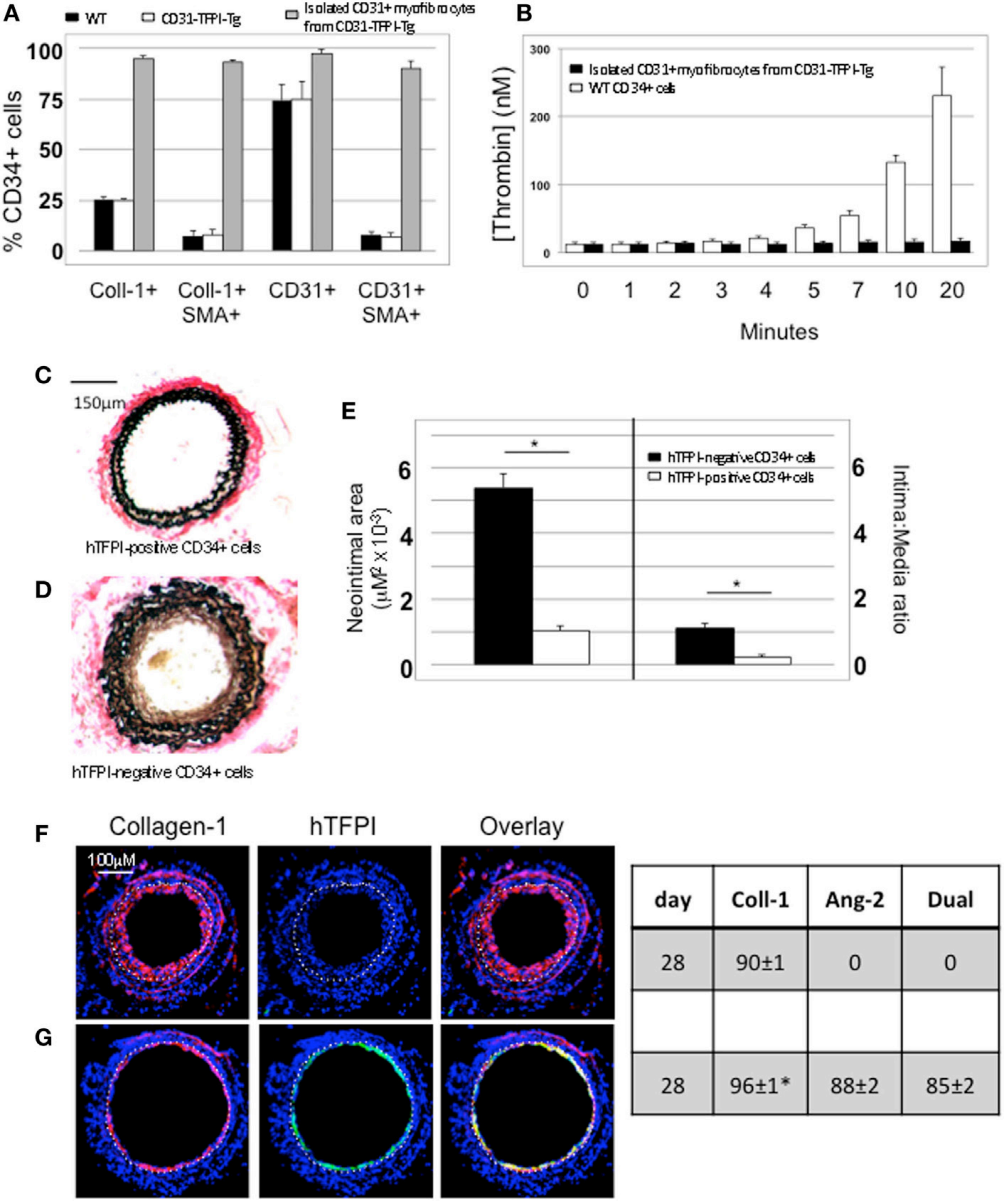
Isolation and characterization of CD31+ myofibrocytes from CD31-TFPI-Tg mice. **(A)** Immunocytofluorescence analysis of CD34+ cells from wild-type (WT) (black bars) or CD31-TFPI-Tg (white bars) mice compared to the purified fraction of CD34+ cells that express human tissue factor pathway inhibitor (hTFPI) on the cell surface (gray bars). Cells were stained with antibodies against anti-smooth muscle actin and either collagen-1 or CD31, and the proportion of cells expressing each was determined. Data are derived from counting at least 100 stained cells from each of five different animals. **(B)** Thrombin generation assay: CD34+ cells from WT (white bars) or purified CD31+ myofibrocytes from CD31-TFPI-Tg (black bars) were incubated with FXa, FVa, and prothrombin as described in Section “Materials and Methods,” and supernatants harvested at the times indicated for analysis of the amount of thrombin generated. **(C–G)** Comparison of the functional impact, after adoptive transfer, of the cell-surface hTFPI+ and hTFPI-negative fractions of CD34+ cells from CD31-TFPI-Tg mice. **(C,D)** Cross sectional images of WT carotid artery 28 days post-injury stained with elastin van Gieson’s stain after adoptive transfer of cell-surface hTFPI+ fraction **(C)** or hTFPI-negative fraction **(D)** on day of injury. **(E)** Neointimal area (left panel) and intima:media ratio (right panel) of vessels taken from WT animals 28 days post-injury after adoptive transfer of 1 × 10^6^ hTFPI-negative (black bars) or hTFPI-positive (white bars) fractions. Data derived from examination of three random sections from five different vessels. *p < 0.01. **(F,G)** Immunohistology of sections through WT injured mouse carotid arteries harvested 28 days post-injury. All sections stained with DAPI (4,6 diamidino-2-phenylindole) nuclear stain (blue) and (red) anti-collagen-1 and (green) anti-hTFPI. Yellow indicates co-localization. The annotated white line defines the junction between neointima and media. Mice received cell-surface hTFPI-negative fraction **(F)** or cell-surface hTFPI+ fraction **(G)** of CD34+ cells on day of injury. Table besides panels **(F,G)** describes summary of staining from all mice (*n* = 6), showing the proportion of the neointimal area that is positive for collagen-1, hTFPI, or both on day 28 (% ± SEM). Data derived from three random sections from each of the arteries from each mouse (see Materials and Methods). Subtracting the proportional area occupied by dual positive cells from the total area occupied by hTFPI+ cells gives the proportional area occupied by non-adoptively transferred collagen-1-positive cells. *cf hTFPI-negative fraction *p* = NS. All experiments repeated at least twice.

We used the constitutive expression of hTFPI to purify myofibrocytes, by magnetic bead separation from CD34+ cells; the isolated myofibrocytes all expressed CD31, SMA, and collagen-1 (Figure 4A), confirming our previous description of myofibrocytes in these mice, but they were angiopoietin-2 negative (see below). To confirm that they expressed cell-surface hTFPI, we showed that the cells failed to promote thrombin generation in the presence of FXa and prothrombin (in contrast to control CD34+ cells from WT mice), consistent with the known inhibitory effect of cell-surface hTFPI, which inhibits FXa (Figure 4B).

Injection of these purified hTFPI+ CD31+ myofibrocytes into injured WT mice inhibited the development of IH (Figures 4C,E), in contrast to injection of the hTFPI-CD31-negative fraction of CD34+ cells, following which IH developed as in WT mice (Figures 4D,E). The injected hTFPI+ CD31+ myofibrocytes were recruited to the site of injury, as evidenced by staining for hTFPI, but they remained angiopoietin-2-negative (Figures 4F,G). These data confirm that inhibition of TF on the adoptively transferred myofibrocytes prevents IH, and that this associates with recruitment of collagen-1+ angiopoietin-2 negative cells to the site of injury.

### Importance of Angiopoietin-2 for Development of IH

#### Association Between IH and Plasma Angiopoietin-2 Levels

**Figure 5:**
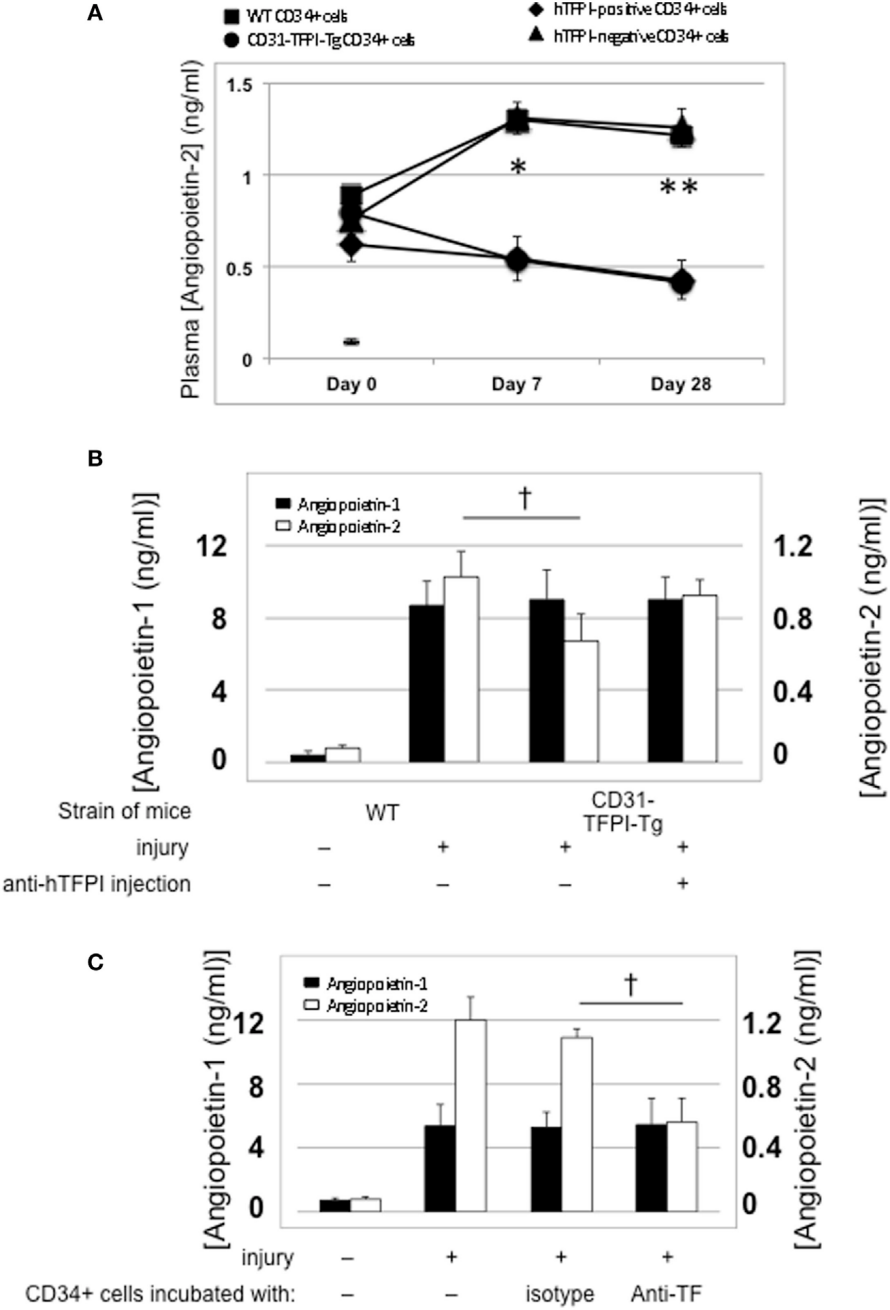
Association between intimal hyperplasia and plasma levels of angiopoietin-2 after wire-induced endoluminal injury. **(A)** Plasma angiopoietin-2 levels in wild-type (WT) mice. Levels in unninjured mice represented by the horizontal bar day 0 only; (squares)—WT CD34+ cells; (circles)—unfractionated CD31-TFPI-Tg CD34+ cells; (diamonds) human tissue factor pathway inhibitor (hTFPI)+ fraction of CD31-TFPI-Tg CD34+ cells; (triangles)—hTFPI-negative fraction of CD31-TFPI-Tg CD34+ cells. *Day 7 *p* < 0.01: WT vs. CD31-TFPI-Tg cells; WT cells vs. hTFPI+ fraction; CD31-TFPI-Tg cells vs. hTFPI− fraction and hTFPI− fraction vs. hTFPI+ fraction. All other comparisons, *p* > 0.05. **Day 28, *p* < 0.01: WT vs. CD31-TFPI-Tg cells; WT cells vs. hTFPI+ fraction; *p* < 0.01: CD31-TFPI-Tg cells vs. hTFPI− fraction and hTFPI− fraction vs. hTFPI+ fraction. All other comparisons, *p* > 0.05. **(B)**: Plasma angiopoietin-1 (black bars, left axis) and angiopoietin-2 (white bars, right axis) levels, in ng/ml, measured on day 3 post-injury. The strain of mice and whether they underwent injury is indicated. CD31-TFPI-Tg mice received anti-hTFPI antibody on day of injury as indicated. ^†^*p* = 0.08. **(C)**: plasma angiopoietin-1 (black bars, left axis), and angiopoietin-2 (white bars, right axis) levels in ng/ml, measured on day 3 post-injury, after adoptive transfer of 1 × 10^6^ WT CD34+ cells that were either pre-incubated with isotype control or an anti-TF antibody immediately prior to injury. A non-injured control is also included for comparison. ^†^*p* < 0.05.

Compared to uninjured mice, the concentration of plasma angiopoietin-2 increased significantly within a few hours post-injury (Figure 5A—day “0”), remained significantly elevated in mice that developed progressive IH, but fell progressively in mice injected with cells from CD31-TFPI-Tg, unless the mice were given an anti-TFPI antibody (Figure 5B), in which case plasma levels normalized, in association with development of IH. Injection of CD34+ cells pre-incubated with anti-TF antibody, which inhibited IH, was also associated with a significant reduction in plasma angiopoietin-2 levels, (Figure 5C). Plasma angiopoietin-1 levels were not influenced under these conditions (Figures 5A,B). These data indicate an association between development of IH and plasma angiopoietin-2 levels.

#### Angiopoietin-2 Expression by Myofibrocytes Is TF and Thrombin-Dependent

**Figure 6:**
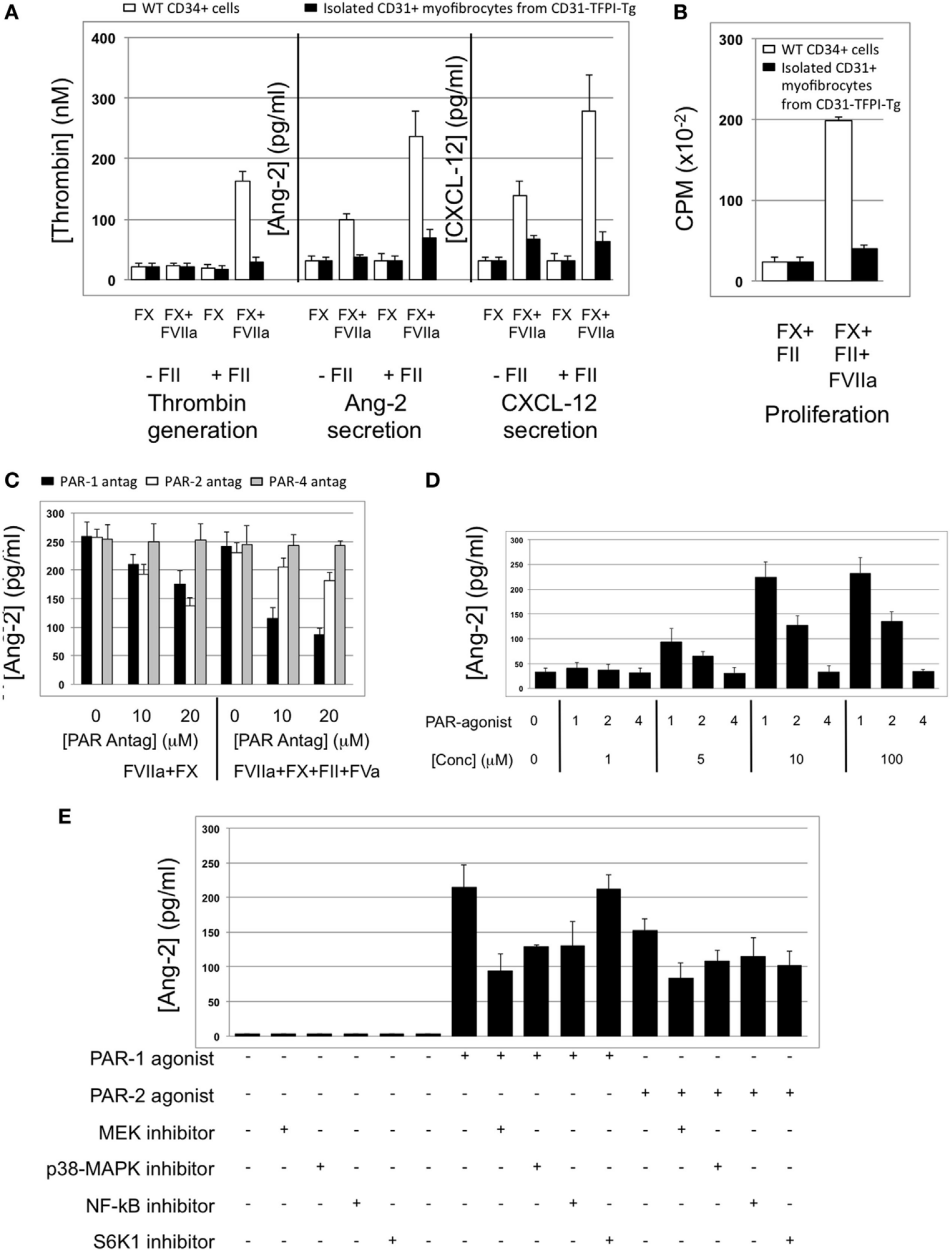
Impact of coagulation proteases on myofibrocyte phenotype. In **(A,B)**, responses of wild-type (WT) CD34+ cells are shown as white bars, whereas isolated CD31+ myofibrocytes from CD31-TFPI-Tg mice are shown as black bars. **(A)** Cells were incubated with FX in presence or absence of FVIIa and FII (prothrombin) plus FVa. Functional tissue factor on WT cells is illustrated by thrombin generation, angiopoietin-2 (Ang-2) secretion, and CXCL-12 secretion. The presence of human tissue factor pathway inhibitor on purified CD31+ myofibrocytes from CD31-TFPI-Tg mice significantly inhibits all three phenotype changes. **(B)** Proliferation, assessed by ^3^H-thymidine incorporation and expressed as counts per minute (CPM) after incubation with FX and FII in presence of FVIIa. **(C)** Angiopoietin-2 secretion by WT CD34+ cells (3 × 10^4^/well) after 24 h incubation with either PAR-1 antagonist (black bars), PAR-2 antagonist (white bars), or PAR-4 antagonist (gray bars) at the indicated concentrations for 30 min before addition of FVIIa with FX (both 10 nM) with or without prothrombin (4 nM) and FVa (6 nM) as indicated. All conditions performed in triplicate wells. Error bars indicate SEM. In comparison of increasing concentrations of antagonists with FVIIa + FX, *p* = 0.027 for PAR2, but *p* = NS for PAR1 and PAR4. In comparison of increasing concentrations of antagonists with FVIIa + FX + FII + FVa, *p* = 0.05 for PAR1, but *p* = not significant (NS) for PAR2 and PAR4. Analysis by one-way ANOVA Kruskal–Wallis test. **(D)** Angiopoietin-2 secretion by WT CD34+ cells (3 × 10^4^/well) after 24 h incubation with PAR-1, -2, or -4 agonists at the indicated concentrations. All conditions performed in triplicate wells. Error bars indicate SEM. *p* = 0.017 for comparisons of increasing concentrations of PAR1 agonist, *p* = 0.012 for PAR2, but *p* = NS for PAR4 agonist. Analysis by one-way ANOVA Kruskal–Wallis test. **(E)** Dissection of signaling pathways involved in angiopoietin-2 secretion by WT CD34+ cells induced by 24 h incubation with 10 mM PAR-1 or -2 agonists. Cells were incubated with the agonists with or without 50 mM mitogen-activated protein kinase inhibitor PD98059, 10 mM p38-MAPK inhibitor SB203580, 20 mM NF-kB inhibitor SN50, or 1 mM of the S6K1 inhibitor as indicated. All conditions performed in triplicate wells. Error bars indicate SEM. *p* = 0.05 for PAR-1 agonist without inhibitor vs. +PD98509 and vs. +SB203580. *p* = > 0.05 all other comparisons. Analysis by Mann–Whitney *T* test. All experiments repeated at least twice.

The majority of CD34+ cells harvested 2 days post-injury express functional TF (5): this was confirmed on WT CD34+ cells by showing that thrombin was generated when cells were incubated with FX and prothrombin when FVIIa was present (Figure 6A). The WT CD34 cells secreted angiopoietin-2 under these conditions, and also proliferated (Figures 6A,B), whereas purified hTFPI-expressing myofibrocytes made no thrombin, angiopoietin-2, and failed to proliferate under identical conditions. Angiopoietin-2 secretion by WT cells under these conditions was predominantly PAR-1-dependent (Figure 6C). However, reduced quantities were detected when WT cells (but not hTFPI+ myofibrocytes) were incubated with FVIIa and FX without prothrombin (Figure 6A), suggesting that some of the secreted angiopoietin-2 was TF- but not thrombin-dependent; under these conditions, PAR-2 signaling was as important as PAR-1 (Figure 6C). Consistent with these data, a PAR-1 agonist induced greater secretion of angiopoietin-2 compared to an equimolar concentration of PAR-2 agonist (Figure 6D). Finally, PAR-1-dependent angiopoietin-2 secretion partially involved signaling through p42/p44 ERK, p38 kinase, and NFkB pathways, but not S6K1 (Figure 6E). PAR-2-induced angiopoietin-2 secretion was also partially inhibited by blocking these same pathways but none of the results reached statistical significance. All these data indicate that on WT cells, TF promotes secretion of angiopoietin-2 and induces proliferation, mainly *via* generation of thrombin through canonical PAR-1 signaling pathways, and that these effects are completely inhibited by the presence of hTFPI on the surface of CD31+ myofibrocytes from CD31-TFPI-Tg mice.

### Thrombin-Dependent Proliferation of Myofibrocytes Is Angiopoietin-2-Dependent

**Figure 7:**
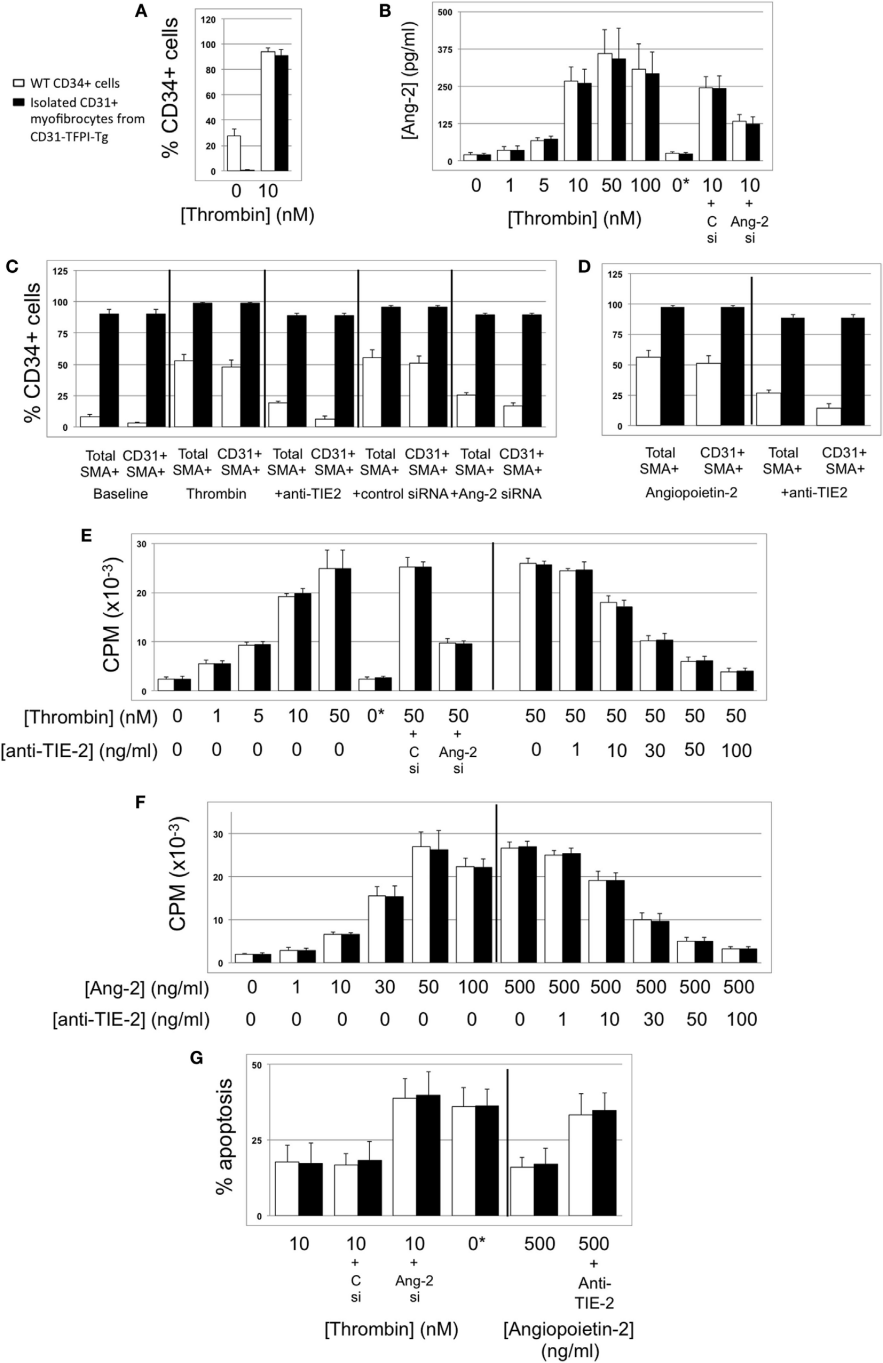
Thrombin-induced proliferation and survival are angiopoietin-2-dependent. In all, responses of wild-type (WT) CD34+ cells are shown as white bars, whereas purified CD31+ myofibrocytes from CD31-TFPI-Tg mice are shown as black bars. 0*Indicates incubation with active site inhibited thrombin. Abbreviations: Csi, control scrambled siRNA; Ang-2si, siRNA specific for angiopoietin-2. **(A)** Expression of angiopoietin-2 at baseline and after incubation with thrombin for 5 days, expressed as the percentage of CD34+cells staining positive in immunocytofluoresence analysis. **(B)** Secretion of angiopoietin-2 into supernatant after 5-day incubation with thrombin at the indicated concentrations, analyzed by ELISA. **(C)** Expression of smooth muscle actin (SMA) and joint expression of CD31 with SMA at baseline and after incubation with thombin for 5 days, either alone or with anti-TIE-2 antibody or siRNA against angiopoietin-2 or control. Data expressed as the percentage of CD34+ cells staining positive in immunocytofluoresence analysis. **(D)** As **(C)** but cells incubated with angiopoietin-2 ± anti-TIE-2 antibody. **(E)** Proliferation, assessed by ^3^H-thymidine incorporation and expressed as counts per minute (CPM) after incubation with thrombin at the indicated concentrations. **(F)** Proliferation, assessed by ^3^H-thymidine incorporation and expressed as CPM after incubation with angiopoietin-2 at the indicated concentrations. **(G)** Degree of apoptosis after incubation with thrombin or angiopoietin-2 at the indicated concentrations. Anti-TIE-2 antibody used at 50 ng/ml. All experiments repeated at least twice.

We have previously shown that a 5-day incubation with thrombin expands the proportion of myofibrocytes present in CD34+ cells from 3 to 80%, *via* a combination of selective outgrowth and reduced apoptosis (5). We repeated these *in vitro* experiments, and showed that the proportion of CD34+ cells expressing angiopoietin-2 increased from 20 to 100%, angiopoietin-2 secretion was induced in a dose-dependent manner, and this was partly inhibited by siRNA against angiopoietin-2 (Figures 7A,B). Similar results were seen when purified CD31+ myofibrocytes from CD31-TFPI-Tg mice were incubated with thrombin, though these cells were angiopoietin-2 negative at baseline. Therefore, these data indicate that the thrombin-induced outgrowth of myofibrocytes associates with angiopoietin-2 expression and secretion by CD31+ myofibrocytes.

These thrombin-induced changes in the composition of the CD34+ population were inhibited by an anti-TIE-2 antibody and by the siRNA against angiopoietin-2 (Figure 7C), suggesting that they were mediated by the angiopoietin-2 secretion induced by thrombin. In support of this, a 5-day incubation with angiopoietin-2 induced the same changes as thrombin, and this was inhibited by an anti-TIE-2 antibody (Figure 7D). Using a combination of siRNA, anti-TIE-2, and incubation with angiopoietin-2 alone, we showed that angiopoietin-2, *via* TIE-2 was responsible for both the enhanced proliferation (Figures 7E,F), and the reduced apoptosis of myofibrocytes induced by thrombin (Figure 7G). As anticipated, the different conditions had little impact on the expression of CD31 or SMA by purified CD31+ myofibrocytes (Figures 7C,D), but these data indicate that the effect of angiopoietin-2 on proliferation and apoptosis was a direct effect on the myofibrocytes.

### IH *In Vivo* Is Angiopoietin-2-Dependent

**Figure 8:**
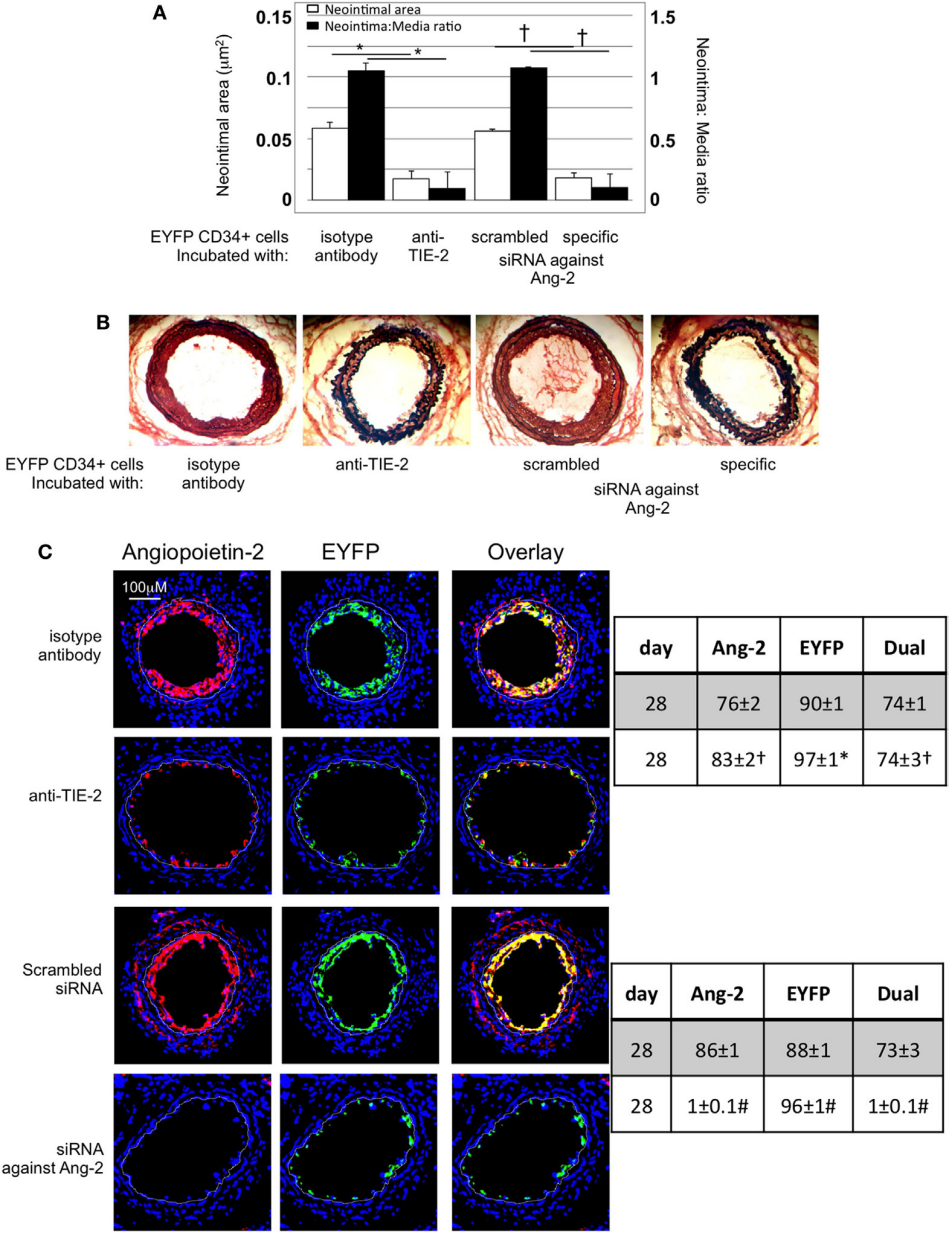
Pre-incubation of enhanced yellow fluorescent protein (EYFP) CD34+ cells with reagents to target angiopoietin-2 prior to adoptive transfer. **(A)** Neointimal area (left axis, white bars) and neointima:media ratio (right axis, black bars) of vessels taken from wild-type (WT) animals 28 days post-injury after adoptive transfer of 1 × 10^6^ EYFP CD34+ cells that were either pre-incubated with isotype control antibody, an anti-TIE-2 antibody, control scrambled siRNA or specific siRNA targeting angiopoietin-2, and administered immediately post-injury. Data derived from examination of three random sections from six different vessels. *p < 0.001, ^†^p < 0.001. **(B)** Cross sectional images of WT carotid artery 28 days post-injury stained with elastin van Gieson’s stain after adoptive transfer of cells pre-incubated with the same reagents as above. **(C)** Panels show immunohistology of sections through injured mouse carotid arteries harvested on day 28 post-injury. All sections stained with DAPI (4,6 diamidino-2-phenylindole) nuclear stain (blue) and anti-angiopoietin-2 (red). The green staining is light emitted by the EYFP cells themselves. Yellow indicates co-localization. The annotated white line defines the junction between neointima and media. Compared to the cells incubated with isotype control antibody, the anti-TIE-2 antibody significantly reduced the neointimal area but did not inhibit angiopoietin-2 expression. Compared to the scrambled siRNA, the specific siRNA targeting angiopoietin-2 significantly reduced the neointimal area in association with abolition of angiopooietin-2 expression by EYFP cells. Tables besides panel **(C)** describe summary of staining from all mice (*n* = 6), showing the proportion of the neointimal area that is positive for EYFP, angiopoietin-2, or both on day 28 (% ± SEM). Data derived from three random sections from each of the arteries from each mouse (see Materials and Methods). Subtracting the proportional area occupied by dual positive cells from the total area occupied by angiopoietin-2+ cells gives the proportional area occupied by non-adoptively transferred angiopoietin-2+ cells. *cf isotype for anti-TIE-2 *p* < 0.001. ^†^cf isotype for anti-TIE-2 *p* = NS. ^#^cf scrambled siRNA *p* < 0.001. Experiments repeated at least twice.

After adoptive transfer, CD34+ cells from EYFP mice incubated with an anti-TIE-2 monoclonal antibody or siRNA against angiopoeitin-2 were recruited to the area of damage, but development of IH was inhibited, compared to controls (Figures 8A,B). Recruited EYFP cells were still angiopoietin-2+ after incubation with the anti-TIE-2 (Figure 8C), and there was evidence of angiopoietin-2 expression by cells other than myofibrocytes at day 28. In contrast, the specific siRNA led to long-term (4-week) inhibition of angiopoietin-2 expression by EYFP+ cells and there was no evidence of angiopoietin-2 expression by other neointimal cells (Figure 8C). This data suggest that angiopoietin-2 secretion by recruited myofibrocytes was responsible for angiopoietin-2 expression by other cells in the vessel wall, but that both angiopoietin-2 *AND* intact TIE-2 signaling on myofibrocytes was needed to develop IH.

### Continued CXCL-12-Dependent Myofibrocyte Recruitment Contributes to IH

#### Association Between CXCL-12 and Angiopoietin-2 *In Vivo*

**Figure 9:**
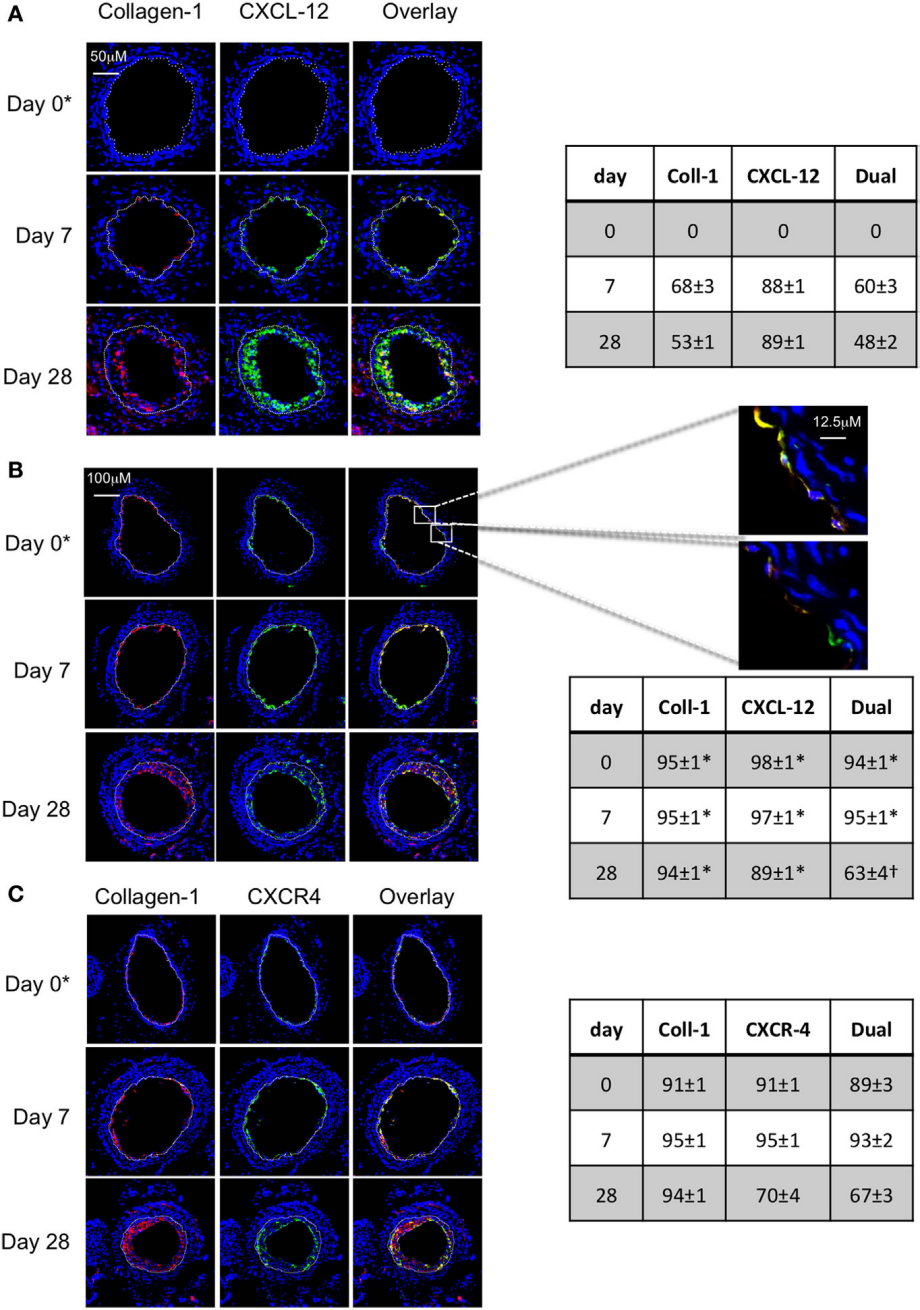
Association between CXCL-12 and intimal hyperplasia (IH). Panels show immunohistology of representative sections through injured mouse carotid arteries harvested on days post-injury as indicated. Sections from same day are consecutive. All sections stained with DAPI (4,6 diamidino-2-phenylindole) nuclear stain (blue) and (red) anti-collagen-1, and (green) anti-CXCL-12 **(A,B)** or CXCR4 **(C)**. Yellow indicates co-localization. The annotated white line defines the junction between neointima and media. **(A)** Injured mice that received no adoptively transferred cells. **(B,C)** Adoptive transfer of wild-type (WT) CD34+ cells to WT mice immediately after post-injury. *Sections harvested within an hour of injury. Expanded areas illustrate that most of the early recruited myofibrocytes are expressing CXCL-12, though there is luminal CXCL-12 that is independent of collagen-1 at early time periods. Tables beside panels describe summary of staining from all mice (*n* = 6), showing the proportion of the neointimal area that is positive for collagen-1, CXCL-12 **(A,B)**, or CXCR4 **(C)** or dual positive on days 0, 7, and 28 (% ± SEM). Data derived from three random sections from each of the arteries from each mouse (see Materials and Methods). *cf no adoptive transfer **(A)** at equivalent time *p* < 0.001. ^†^cf no adoptive transfer **(A)** at equivalent time *p* = 0.02. Experiments repeated at least twice.

In WT mice, a significant proportion of myofibrocytes co-expressed CXCL-12 throughout the development of IH (Figure 9A), though as with angiopoietin-2 expression, a significant proportion of collagen-1-negative intimal cells also expressed CXCL-12. A similar pattern of staining was seen after adoptive transfer of WT cells (Figure 9B). At the earliest time point, it was apparent that some of the luminal CXCL-12 staining appeared to be independent of collagen-1 (Figure 9B). Most neointimal cells also expressed CXCR4 throughout the period post-injury (Figure 9C).

**Figure 10:**
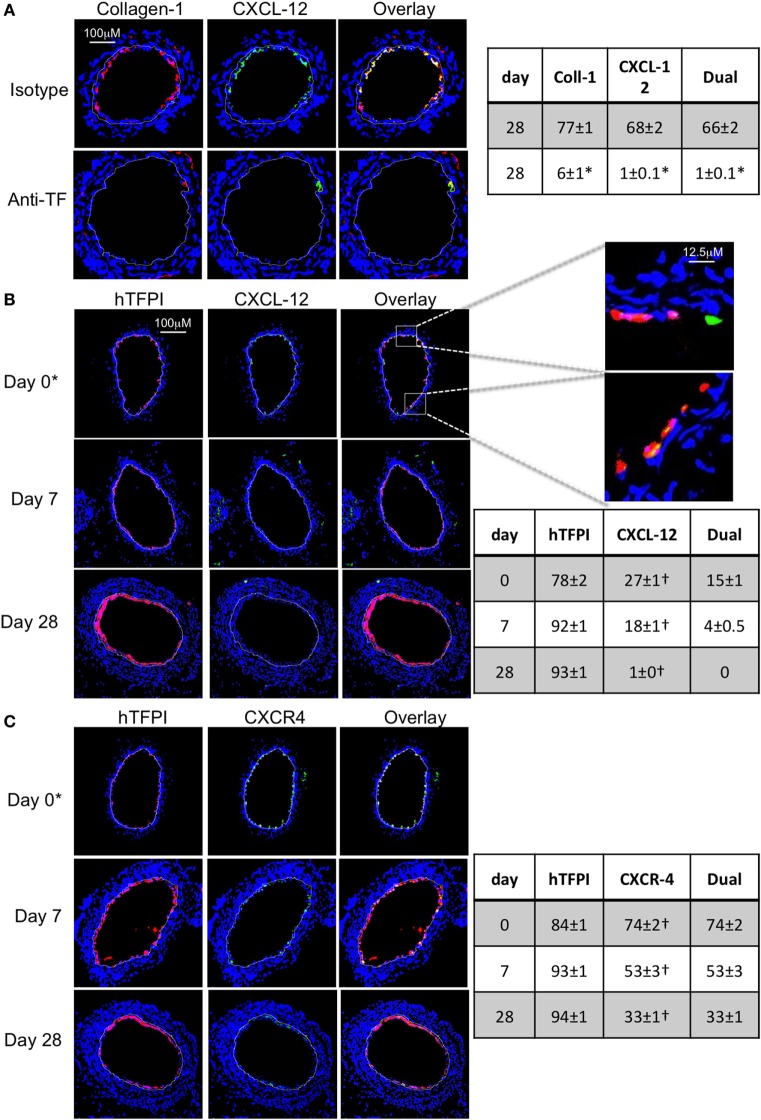
Recruitment after adoptive transfer with inhibition of tissue factor (TF). Panels show immunohistology of representative sections through injured mouse carotid arteries harvested on days post-injury as indicated. Sections from same day are consecutive. All sections stained with DAPI (4,6 diamidino-2-phenylindole) nuclear stain (blue) and (red) anti-collagen-1 **(A)** or human tissue factor pathway inhibitor (hTFPI) **(B,C)**, and (green) anti-CXCL-12 **(A,B)** or CXCR4 **(C)**. Yellow indicates co-localization. The annotated white line defines the junction between neointima and media. **(A)** Day 28 sections, in wild-type (WT) mice adoptively transferred CD34+ cells pre-incubated with istotype control or anti-TF antibody. **(B,C)** Adoptive transfer of purified CD31+ myofibrocytes from CD31-TFI-Tg mice to WT mice immediately post-injury. *Sections harvested within an hour of injury. Expanded areas illustrate that some of the early luminal CXCL-12 staining is independent of recruited hTFPI+ cells. Table besides **(A)** describes summary of staining from all mice (*n* = 6), showing the proportion of the neointimal area that is positive for collagen-1, CXCL-12, or both on day 28 (% ± SEM), whereas those besides **(B,C)** described proportion that is positive for hTFPI, CXCL-12 **(B)**, CXCR4 **(C)**, or dual positive on days 0, 7, and 28. Data derived from three random sections from each of the arteries from each mouse (see Materials and Methods). *cf isotype for anti-TF *p* < 0.001. ^†^cf adoptive transfer of WT cells (Figures 9B,C) at equivalent times *p* < 0.001. Experiments repeated at least twice.

In mice that received CD34+ cells treated with an anti-TF antibody, CXCL-12 expression in the neointima was abolished, but with the caveat mentioned above that few myofibrocytes were recruited (Figure 10A). After adoptive transfer of CD34+ cells from CD31-TFPI-Tg mice, most of the hTFPI+ cells recruited to the site of injury were CXCL12 negative (Figure 10B), and all became CXCL-12-negative with increasing time from the injury. As with WT cells, early (but not late) luminal CXCL-12-expression appeared to be independent of the adoptively transferred cells (Figure 10B). CXCR4 was also seen co-localized with hTFPI at early time points (Figure 10C), suggesting that the initial myofibrocytes recruited immediately after injury expressed CXCR4, but expression faded with time, so that neointimal cells 28 days post-injury were mostly CXCR4-negative.

**Figure 11:**
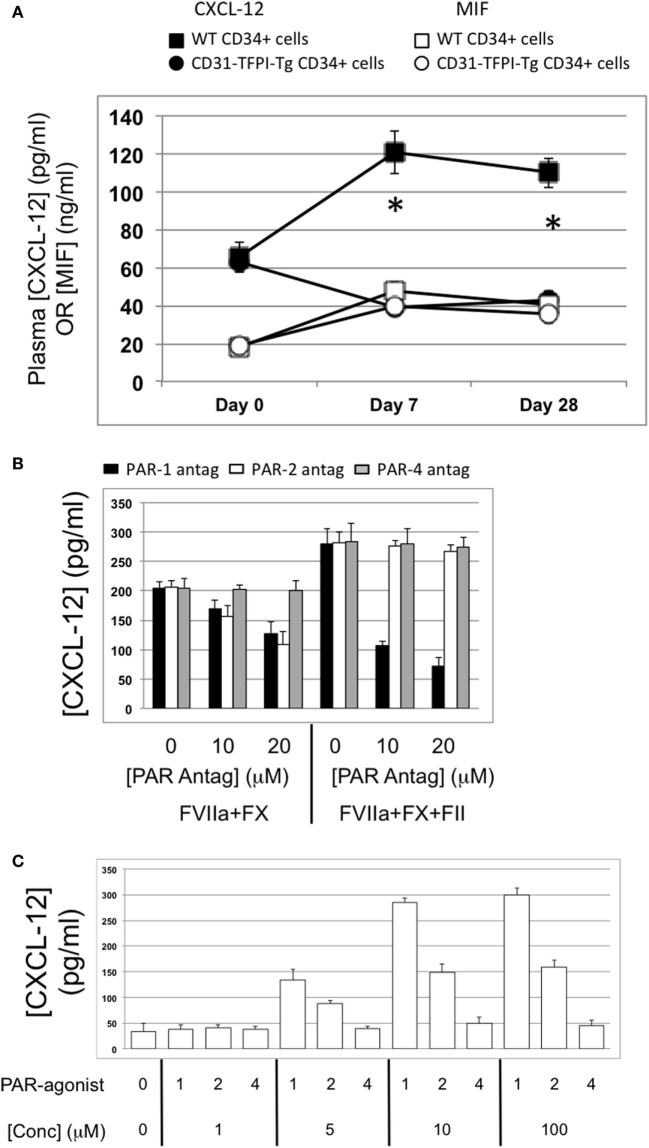
Association between plasma CXCL-12 and intimal hyperplasia (IH): CXCL-12 expression is thrombin and angiopoietin-2-dependent. **(A)** Plasma CXCL-12 (black filled) and migration inhibitory factor (MIF) (white filled) levels post-injury in wild-type (WT) recipients of WT (squares) or CD31-TFPI-Tg (circles) CD34+ cells. *Day 7 and day 28 *p* < 0.005 WT vs. CD31-TFPI-Tg for CXCL-12. *p* = NS for all comparisons of MIF. **(B)** CXCL-12 secretion by WTCD34+ cells (3 × 10^4^/well) after 24 h incubation with either PAR-1 antagonist (black bars), PAR-2 antagonist (white bars), or PAR-4 antagonist (gray bars) at the indicated concentrations for 30 min before addition of FVIIa with FX (both 10 nM) with or without prothrombin (4 nM) and FVa (6 nM) as indicated. All conditions performed in triplicate wells. Error bars indicate SEM. **(C)** Angiopoietin-2 secretion by WT CD34+ cells (3 × 10^4^/well) after 24-h incubation with PAR-1, -2, or -4 agonists at the indicated concentrations. All conditions performed in triplicate wells. Error bars indicate SEM. Data shown in **(B,C)** is from WT CD34+ cells. Abbreviations: Csi, control siRNA; Ang-2si, siRNA specific for angiopoietin-2. Experiments repeated at least twice.

As for plasma angiopoietin-2 levels, post-injury plasma CXCL-12 levels increased over the first week and remained high in mice that received WT CD34+ cells, but reduced in mice receiving CD34+ cells from CD31-TFPI-Tg mice (Figure 11A). These differences were specific, as levels of another TF and thrombin-dependent chemokine macrophage migration inhibitory factor (12) were similar in both groups of mice (Figure 11A). These data suggested that, as for angiopietin-2, levels of CXCL-12 in the blood correlated closely with the development of IH.

### Late Myofibrocyte Recruitment Is CXCL-12-Dependent

**Figure 12:**
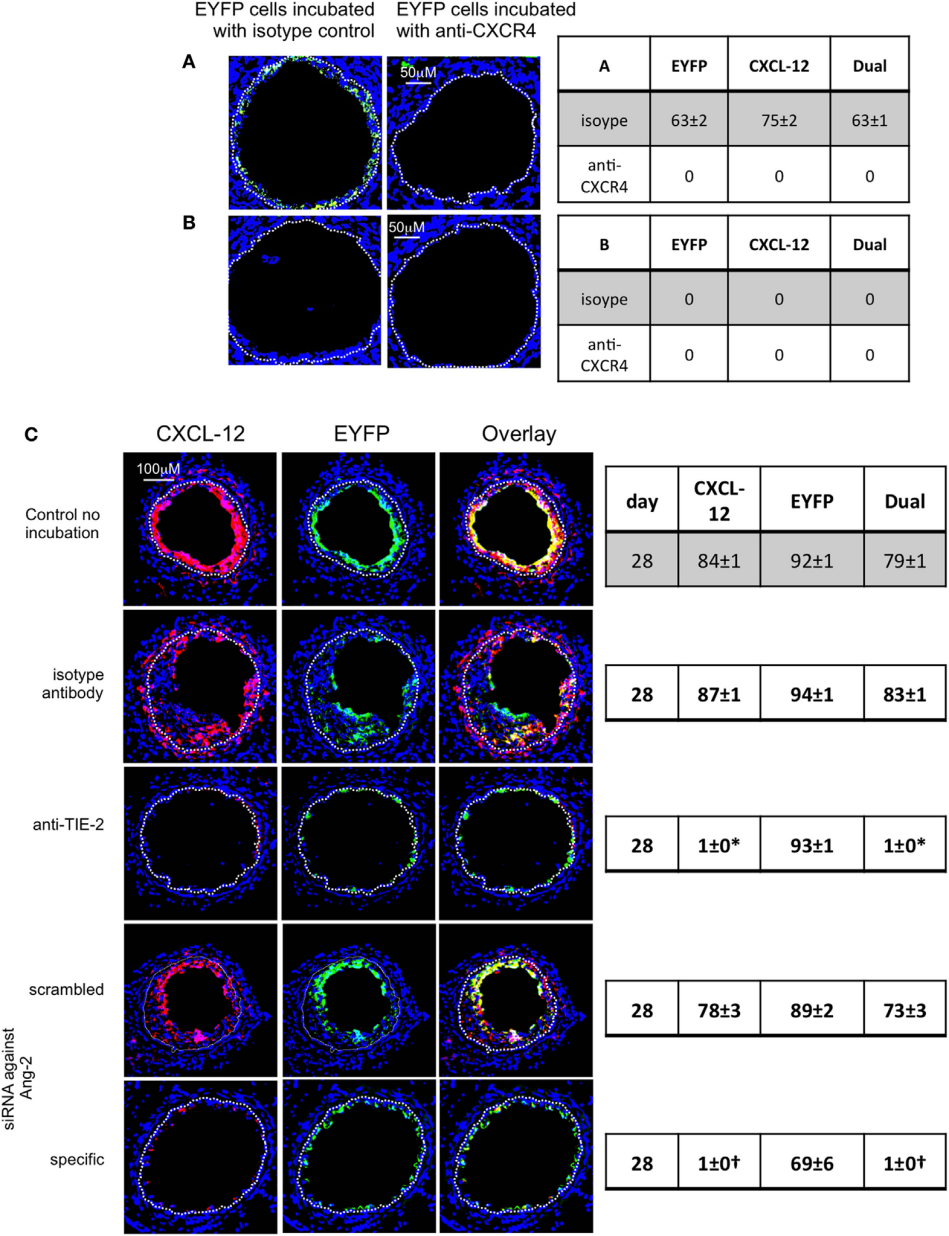
Pre-incubation of enhanced yellow fluorescent protein (EYFP) CD34+ cells with reagents to target CXCR4 or angiopoietin-2 prior to adoptive transfer. Panels show immunohistology of consecutive sections through injured mouse carotid arteries harvested on day 28 post-injury. All sections stained with DAPI (4,6 diamidino-2-phenylindole) nuclear stain (blue) and anti-CXCL-12 (red). The green staining is light emitted by the EYFP cells themselves. Yellow indicates co-localization. The annotated white line defines the junction between neointima and media. **(A,B)** Injured wild-type (WT) mice were adoptively transferred with CD34+ cells on day of injury. In **(A)**, the cells came from WT mice. In **(B)**, the cells came from CD31-TFPI-Tg mice. 1 week later, both groups of mice received a second adoptive transfer of EYFP CD34+ cells that were pre-incubated with either isotype control antibody or anti-CXCR4 as indicated. Sections were harvested 1 week later (day 14 post-injury). The annotated white line defines the junction between neointima and media. The anti-CXCR4 prevented late recruitment of adoptively transferred CD34+ cells in **(A)**, but in **(B)**, there is no late recruitment of adoptively transferred EYFP cells, even those pre-incubated with control antibody. **(C)** Adoptive transfer of EYFP CD34+ cells to injured WT mice at the time of injury. Cells first incubated with an anti-TIE-2 or isotype control, or siRNA targeting angiopoietin-2 or scrambled control. Compared to the control cells and those incubated with isotype control antibody, the anti-TIE-2 antibody significantly reduced the neointimal area but did not inhibit angiopoietin-2 expression. Compared to the scrambled siRNA, the specific siRNA targeting angiopoietin-2 significantly reduced the neointimal area in association with abolition of angiopoietin-2 expression cells. Tables besides panels describe summary of staining from all mice (*n* = 6), showing the proportion of the neointimal area that is positive for CXCL-12, EYFP, or both on day of examination (% ± SEM). Data derived from three random sections from each of the arteries from each mouse (see Materials and Methods). *cf isotype for anti-TIE-2 *p* < 0.001. ^†^cf scrambled siRNA *p* < 0.001. Experiments repeated at least twice.

To demonstrate that myofibrocyte CXCL-12 was relevant for continued myofibrocyte recruitment, some mice received an injection of CD34+ cells from EYFP mice, 1 week after injection of WT cells. The EYFP CD34+ cells were pre-incubated with either an anti-CXCR4 antibody or an isotype control. Whereas EYFP cells exposed to the isotype control were found in the intima 1 week later, no EYFP+ cells exposed to the anti-CXCR4 were recruited (Figure 12A), strongly suggesting that the late recruitment of cells to the neointima was CXCR4-dependent. When these mice had first received cells from CD31-TFPI-Tg mice, there was no evidence of recruitment of EYFP cells when vessels were examined 1 week later (Figure 12B), even in control cells, indicating that the absence of CXCL-12 secretion by neointimal hTFPI+ myofibrocytes prevented continued myofibrocyte recruitment.

### CXCL-12 Secretion by Myofibrocytes Is TF and Angiopoietin-2-Dependent

**Figure 13:**
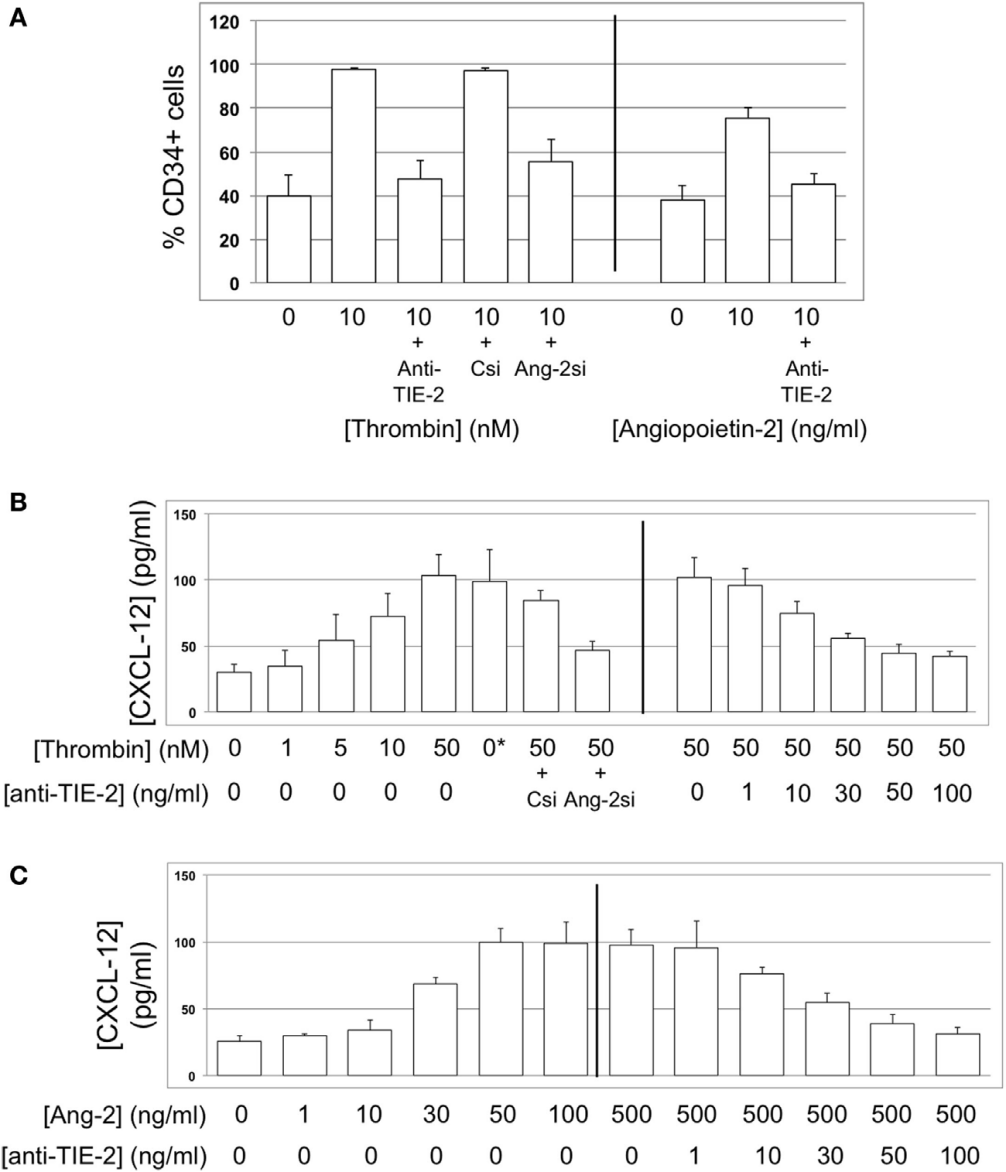
CXCL-12 expression is thrombin and angiopoietin-2-dependent. **(A)** Expression of CXCL-12 at baseline and after incubation with thrombin or angiopoietin-2 for 5 days, expressed as the percentage of cells staining positive in immunocytofluorescence analysis. Anti-TIE-2 antibody used at 50 ng/ml. **(B)** Secretion of CXCL-12 into supernatant after 5 day incubation with thrombin at the indicated concentrations, analyzed by ELISA. **(C)** Secretion of CXCL-12 into supernatant after 5 day incubation with angiopoietin-2 at the indicated concentrations, analyzed by ELISA. All data shown from WT CD34+ cells. Abbreviations: *Csi*, control siRNA. *Ang-2si*, siRNA specific for angiopoietin-2. Experiments repeated at least twice.

To explore the link between TF, thrombin, angiopoietin-2, and CXCL-12, we incubated WT CD34 cells with FX, FVIIa, and prothrombin; these cells secreted CXCL-12, whereas purified hTFPI-expressing myofibrocytes made little under identical conditions (Figure 6A). CXCL-12 from WT CD34+ cells under these conditions was predominantly PAR-1-dependent (Figure 11B). Reduced quantities were secreted when WT cells were incubated with FVIIa and FX without prothrombin (Figure 6A), and under these conditions, PAR-2 signaling was as important as PAR-1 (Figure 11B). However, as for angiopoietin-2, a PAR-1 agonist induced greater secretion of CXCL-12 compared to an equimolar concentration of PAR-2 agonist, confirming the predominance of PAR-1 (Figure 11C). After incubation with thrombin, the proportion of WT CD34+ cells expressing CXCL-12 increased from 40 to 100% (Figure 13A) and induced CXCL-12 secretion (Figure 13B). These thrombin-dependent effects were inhibited by an inhibitory anti-TIE-2 antibody and by the siRNA against angiopoietin-2, and incubation with angiopoietin-2 induced the same changes, also in a TIE-2-dependent manner (Figure 13C).

These data indicate that, alongside proliferation and survival, the CXCL-12 secretion induced in myofibrocytes by TF-induced activation of coagulation proteases was mostly thrombin and PAR-1-dependent, and was mediated *via* angiopoietin-2 secretion and interaction with TIE-2.

Finally, to confirm the link between angiopoietin-2 and CXCL-12 *in vivo*, we demonstrated that adoptively transferred EYFP+ CD34+ cells recruited to the site of injury expressed CXCL-12 (Figure 12C). However, EYFP CD34+ cells treated prior to transfer with either an anti-TIE-2 monoclonal antibody or siRNA against angiopoietin-2 made little CXCL-12 compared to controls (Figure 8 NEW Figure 12C), supporting the *in vitro* findings that CXCL-12 production was angiopoietin-2 and TIE-2-dependent.

## Discussion

Intimal hyperplasia following vascular injury occurs because of a progressive accumulation of cells expressing SMA in the neointima, which together with vascular remodeling, causes stenosis and ischemia of downstream tissues. Although the origin of neointimal SMA+ cells in some models of IH remains contentious, in our early work with both wire-induced injury and allogeneic aortic transplant models (4), we showed that a BM-derived cell type, subsequently identified as a myofibrocyte, had a critical influence on the development of IH (5, 6). On discovering that these cells were mobilized into the circulation immediately post-injury, we developed two adoptive transfer models, whereby CD34+ cells isolated from post-injury donor mice are transferred to host mice either on the day of host mouse injury or 7 days post-allogeneic transplantation (4, 5, 8). Under both these conditions, the transferred donor cells are recruited early to the sites of injury.

These adoptive transfer models, which we show here reflect closely the disease that develops in WT mice, offer controlled experimental systems in which to study the cellular basis of IH. Transferred CD34+ cells from either WT or CD31-TFPI-Tg mice contain approximately 25–50% fibrocytes (CD34+, collagen-1+ CD45+), 20% of which are myofibrocytes (SMA+) and some of these (i.e., approximately 5% of the transferred CD34+ cells) are of the phenotype (CD31+) that gets recruited to the vessel wall (5, 8). We demonstrate here that these recruited cells, as well as undergoing hyperplasia at the site of injury, also induce changes in host vessel wall cells. Our findings, therefore, do not challenge or contradict the evidence that other cells types, such as those derived from smooth muscle cells, play an important role in IH (13, 14), but they do suggest that the involvement of other cell types may be orchestrated or regulated by myofibrocytes.

Fibrocytes were re-defined in the modern era in a mouse model of wound healing as myeloid cells expressing CD34 and collagen-1. SMA expression has been described (“myofibrocytes”), in association with IH in ovine (15) and rat models. Fibrocytes expressing low levels of CD31 (16) have been described but their function has not been defined.

Having previously defined the significance of TF expression by myofibrocytes, in this new work we confirm that IH is TF-dependent using two orthogonal approaches. First, we capitalized on the fact that, for a reason not yet clarified, in CD31-TFPI-Tg mice, CD31+ myofibrocytes are the only CD34+ subpopulation that express the hTFPI fusion protein on their cell surface, allowing us to isolate them from other CD34+ subpopulations, and confirm that adoptive transfer of this subset alone was able to completely inhibit IH, whereas the remaining, cell surface hTFPI-negative fractions failed to inhibit, thus establishing that the protected phenotype of parental transgenic mice was due entirely to cell surface expression of hTFPI (and inhibition of TF) on this subset. Second, incubation of WT CD34+ cells with an anti-TF antibody prior to adoptive transfer completely inhibited subsequent development of IH, confirming that preventing coagulation protease activation on the surface of the injected CD34+ cells was sufficient to prevent IH. However, inhibition of TF on all subsets of CD34+ cells appeared to have a significant effect on the recruitment of collagen-1+ cells. These two distinct effects of TF inhibition are completely consistent with our previous findings (8).

**Figure 14:**
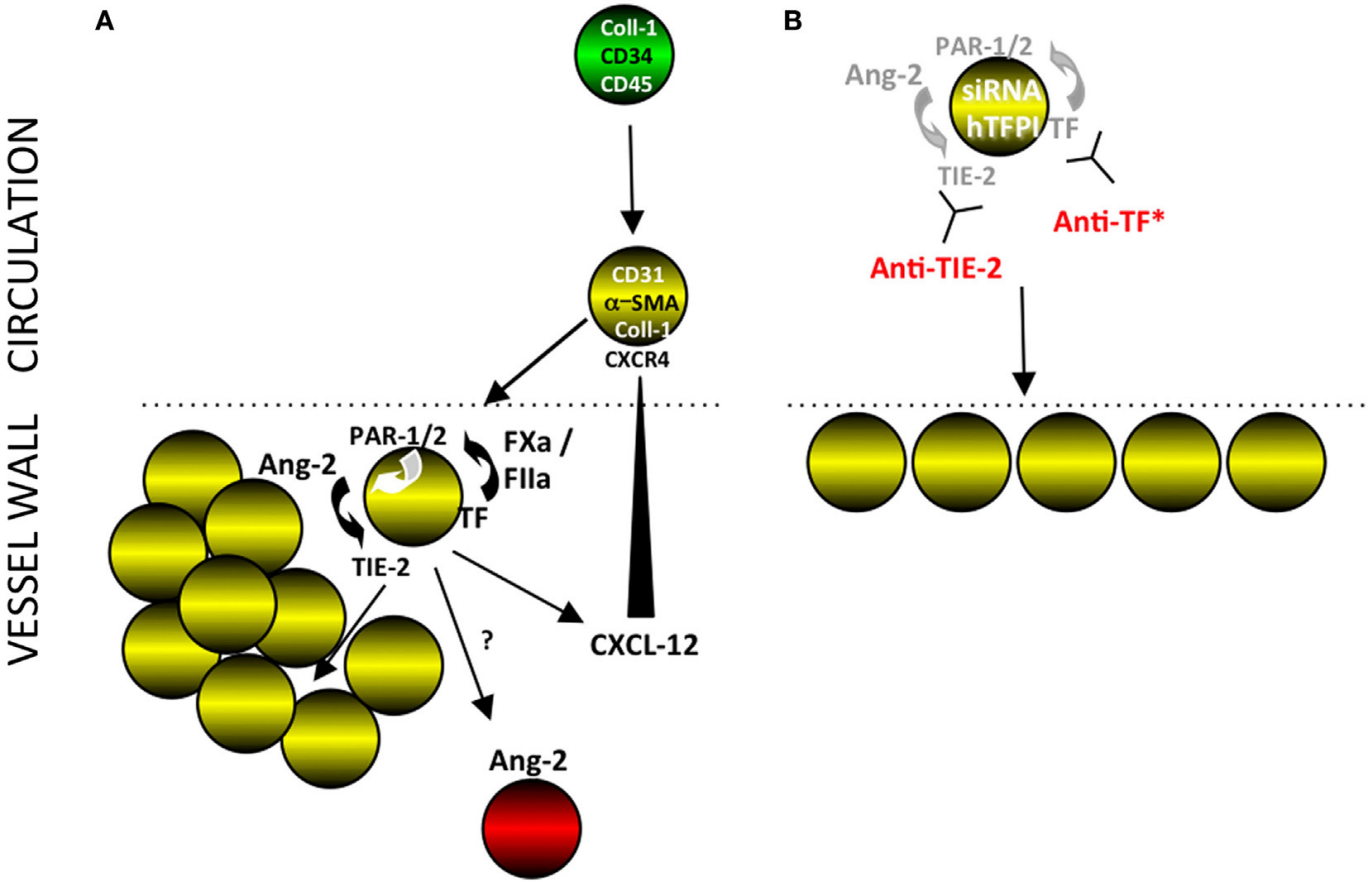
Interpretation of our findings. **(A)** Post-endoluminal injury, fibrocytes (green) are mobilized into the circulation, a subpopulation of which express smooth muscle actin (SMA) (“myofibrocytes”-yellow cells) and CD31 and these are recruited to the site of injury, *via* CXCR4 from CXCL-12 released from luminal platelets (data not shown). These myofibrocytes express tissue factor (TF), which generates FXa and thrombin (FIIa), which can signal through PARs-1 and -2 on the cell surface. These result in the secretion of angiopoietin-2, which through cell surface TIE-2, causes proliferation and secretion of CXCL-12, which can attract more myofibrocytes from the circulation. The myofibrocytes also orchestrate expression of angiopoietin-2 by other cells within the vessel wall, by a mechanism not elucidated. **(B)** Inhibition of TF on the myofibrocytes, either by cell surface expression of a transgenic human tissue factor pathway inhibitor (hTFPI) fusion protein, or by an anti-TF antibody inhibits angiopoietin-2 secretion and results in a small neointima, consisting of non-proliferating myofibrocytes. An siRNA to angiopoietin-2 or a blocking anti-TIE-2 antibody has the same effect, illustrating the key importance of the angiopoietin-2/TIE axis for the development of intimal hyperplasia. *Treatment of whole CD34+ cells with an anti-TF inhibits intimal hyperplasia (IH) but also prevents significant recruitment of myofibrocytes, in a similar way as expressing hTFPI fusion protein on SMA+ fibrocytes (8).

Furthermore, we defined the mechanism through which TF induces hyperplasia of myofibrocytes *in vitro*. TF with FVIIa and FX upregulated production of angiopoietin-2, primarily *via* thrombin generation, which then directly induced TIE-2-dependent proliferation, reduced spontaneous apoptosis, and induced CXCL-12 production. The relevance of these for IH *in vivo* was directly demonstrated by showing that prior treatment of adoptively transferred CD34+ cells with either an anti-TIE-2 or siRNA to angiopoietin-2 completely inhibited the development of IH (see Figure 14). Our experimental approach, being able to isolate CD31+ myofibrocytes from CD31-TFPI-Tg mice, allowed us to show that the effect of thrombin and angiopoietin-2 was a direct effect on the myofibrocytes, and did not involve responses from other CD34+ subpopulations.

Angiopoietin-2 was first defined at sites of vascular remodeling (17) where it disrupted angiogenesis, and is now thought to act as an autocrine factor, sensitizing (and destabilizing) endothelium to activation by other stimuli. Physiologically, these effects are counteracted by angiopoietin-1, made by pericytes *in situ*, which play an important role in maintaining endothelial cell homeostasis (18). These antagonistic effects of angiopoietins-1 and -2 are mediated through their mutual receptor TIE-2 and are thought to be important for maintenance of vascular quiescence (19). Angiopoietin-2 also has direct effects, *via* TIE-2 signaling on some leukocytes (20), including acting as a chemoattractant for monocytes (21) and neutrophils (22) modifying the cytokine profiles of TIE-2 expressing monocytes (23, 24) and has been recently shown to mediate the thrombin-mediated adhesion of monocytes to EC (25). These effects of angiopoietin-2 have been linked to IH in several models (26, 27), and protection against IH linked to antagonism by angiopoietin-1 (28), but our findings provide the first detailed understanding, on the molecular and cellular level, of how angiopoiein-2 regulates the development of IH by linking coagulation proteases with chemokine signaling.

Angiopoietin-2 is constitutively expressed by EC, but expression by other cell types has been described, including myofibroblasts in healing wounds (29) and monocytes during sepsis (30), so our description of a subset of fibrocytes making angiopoietin-2 after stimulation with coagulation proteases is consistent with these previous reports. The association between plasma angiopoietin-2 levels and development of IH is consistent with the neointimal fibrocytes being the main source of plasma angiopoietin-2 in this model.

Early recruitment of myofibrocytes to the site of injury in this model of IH is known to involve CXCL-12, released from platelets that rapidly adhere to areas of endothelial denudement (31, 32). In light of our previous demonstration that platelets adhere rapidly to the site of injury for up to 3 days in this model (6), our new data showing luminal CXCL-12 staining, independently of collagen-1, in sections from a few hours post-injury, is consistent with expression by platelets. We also previously showed that labeled CD34+ cells injected up to 2 weeks after injury were still recruited to the neointima, suggesting that continued recruitment of CD31+ myofibrocytes from the circulation may contribute to the progressive IH. Our new data are consistent with other reports describing the importance of CXCL-12/CXCR4 for progressive IH (33, 34). We have confirmed that this continued recruitment required CXCR4 on the recruited cells and ongoing CXCL-12 expression by neointimal cells, which was associated with sustained levels of circulating CXCL-12 post-injury. *In vivo*, expression of hTFPI on CD31+ myofibrocytes inhibited angiopoietin-2 and CXCL-12 expression and prevented continued recruitment. *In vitro*, CXCL-12 induced by TF or thrombin was mediated by angiopoietin-2 *via* TIE-2.

In summary, we have provided an in-depth analysis of the importance of TF for progressive IH in a model of endoluminal injury in mice, first verifying the importance of TF for hyperplasia of CD31+ myofibrocytes and then defining the critical importance of angiopoietin-2 production, which appears necessary for proliferation, enhanced survival, and continued recruitment of neointimal cells. These data provide important novel insights into the pathophysiology of IH and indicate new translational targets for investigation and potential exploitation in human disease.

## Ethics Statement

This study was carried out in accordance with the recommendations of Ethical Review Committee of King’s College London. The protocols were approved by the UK Home Office.

## Author Contributions

DC, E-LT, L-LW, NM, PD, and YL performed the experimental work. KL, DK, JM, and AD provided intellectual input, and wrote and edited the manuscript. AD conceived the work.

## Conflict of Interest Statement

The authors declare that the research was conducted in the absence of any commercial or financial relationships that could be construed as a potential conflict of interest. The reviewer IM and handling Editor declared their shared affiliation.
