# Supplementary material for: Inhibition of Angiopoietin-2 Production by Myofibrocytes Inhibits Neointimal Hyperplasia After Endoluminal Injury in Mice
Source: Front Immunol. 2018 Jul 2;9:1517. doi: 10.3389/fimmu.2018.01517 (PMC6036182; doi:10.3389/fimmu.2018.01517)
Supplement: Supplementary file 1 [file data_sheet_1.PDF]

Supplementary Figure 1

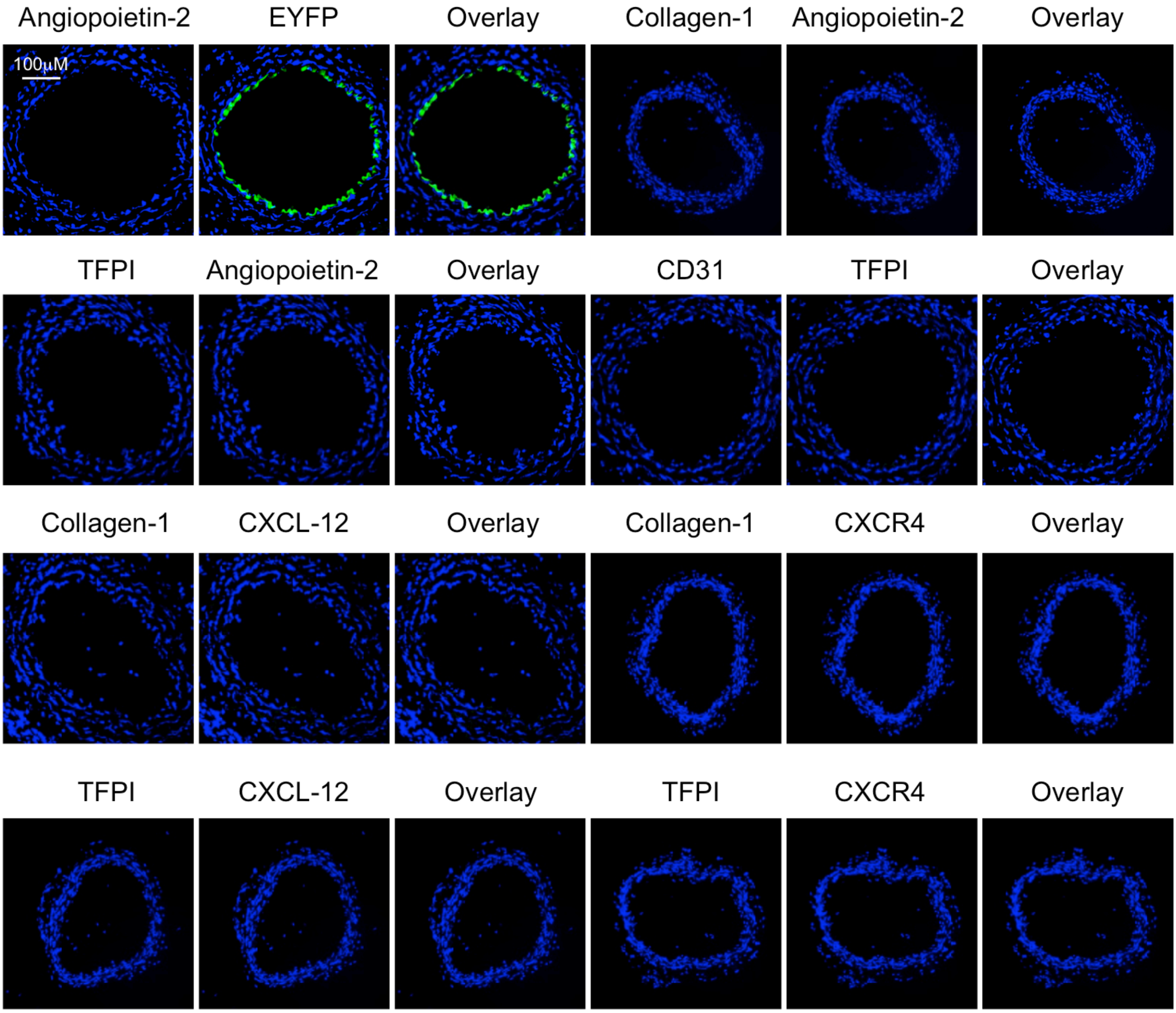

Figure Legend

Supplementary figure 1: Isotype controls

Slide shows isotype control staining for all the specific antibodies used in immunofluorescence analyses in this study
